# Supplementary material for: Revisiting the African mtDNA landscape through complete mitochondrial genomes
Source: Commun Biol. 2026 Jun 3;9:754. doi: 10.1038/s42003-026-10330-9 (PMC13234189; doi:10.1038/s42003-026-10330-9)
Supplement: Supplementary file 2 — Description of Additional Supplementary Files [file 42003_2026_10330_MOESM2_ESM.pdf]

Description of Additional Supplementary File

File name: Supplementary Data 1
Description: Metadata for full mitochondrial database.

File name: Supplementary Data 2
Description: The source data behind the graphs in the paper.

File name: Supplementary Data 3
Description: NEXUS tree topology of African mitochondrial haplogroups.

File name: Supplementary Data 4
Description: NEXUS tree topology of L0k sequences.

File name: Supplementary Data 5
Description: NEXUS tree topology of L0f sequences.

File name: Supplementary Data 6
Description: NEXUS tree topology of L0d sequences.

File name: Supplementary Data 7
Description: NEXUS tree topology of L0a sequences.

File name: Supplementary Data 8
Description: NEXUS tree topology of L1c sequences.

File name: Supplementary Data 9
Description: NEXUS tree topology of L2a sequences.

File name: Supplementary Data 10
Description: NEXUS tree topology of L3e sequences.
