# Supplementary material for: Revisiting the African mtDNA landscape through complete mitochondrial genomes
Source: Commun Biol. 2026 Jun 3;9:754. doi: 10.1038/s42003-026-10330-9 (PMC13234189; doi:10.1038/s42003-026-10330-9)
Supplement: Supplementary file 13 — Reporting summary [file 42003_2026_10330_MOESM13_ESM.pdf]

nature portfolio

Corresponding author(s): Carina Schlebusch

Last updated by author(s): Apr 10, 2026

Reporting Summary

Nature Portfolio wishes to improve the reproducibility of the work that we publish. This form provides structure for consistency and transparency in reporting. For further information on Nature Portfolio policies, see our Editorial Policies and the Editorial Policy Checklist.

Statistics

For all statistical analyses, confirm that the following items are present in the figure legend, table legend, main text, or Methods section.

n/a	Confirmed	
☐	☒	The exact sample size (*n*) for each experimental group/condition, given as a discrete number and unit of measurement
☒	☐	A statement on whether measurements were taken from distinct samples or whether the same sample was measured repeatedly
☒	☐	The statistical test(s) used AND whether they are one- or two-sided<br>*Only common tests should be described solely by name; describe more complex techniques in the Methods section.*
☒	☐	A description of all covariates tested
☒	☐	A description of any assumptions or corrections, such as tests of normality and adjustment for multiple comparisons
☐	☒	A full description of the statistical parameters including central tendency (e.g. means) or other basic estimates (e.g. regression coefficient) AND variation (e.g. standard deviation) or associated estimates of uncertainty (e.g. confidence intervals)
☒	☐	For null hypothesis testing, the test statistic (e.g. *F*, *t*, *r*) with confidence intervals, effect sizes, degrees of freedom and *P* value noted<br>*Give P values as exact values whenever suitable.*
☐	☒	For Bayesian analysis, information on the choice of priors and Markov chain Monte Carlo settings
☒	☐	For hierarchical and complex designs, identification of the appropriate level for tests and full reporting of outcomes
☒	☐	Estimates of effect sizes (e.g. Cohen's *d*, Pearson's *r*), indicating how they were calculated

Our web collection on statistics for biologists contains articles on many of the points above.

Software and code

Policy information about availability of computer code

Data collection	All data generated or analyzed during this study is included in this published article, its supplementary information files and publicly available repositories. The generated mitochondrial data will be available for academic research use through the NCBI database.
Data analysis	Scripts are available on freely available on Zenodo (https://doi.org/10.5281/zenodo.18847351) and Github (https://github.com/imkelankheet/Full_mitochondrial_genomes) and state the software, including software version used for the analyses.

For manuscripts utilizing custom algorithms or software that are central to the research but not yet described in published literature, software must be made available to editors and reviewers. We strongly encourage code deposition in a community repository (e.g. GitHub). See the Nature Portfolio guidelines for submitting code & software for further information.

Data

Policy information about availability of data

All manuscripts must include a data availability statement. This statement should provide the following information, where applicable:

- Accession codes, unique identifiers, or web links for publicly available datasets
- A description of any restrictions on data availability
- For clinical datasets or third party data, please ensure that the statement adheres to our policy

All data generated or analyzed during this study is included in this published article, its supplementary information files and publicly available repositories. The

generated mitochondrial data will be available for academic research use through the NCBI database with accession numbers PV558957–PV560130, and PX394655 and PX394656. Fastq files will be available on ENA with study number PRJEB108938, accession numbers ERS29408145–ERS29409320.

Research involving human participants, their data, or biological material

Policy information about studies with human participants or human data. See also policy information about sex, gender (identity/presentation), and sexual orientation and race, ethnicity and racism.

Reporting on sex and gender	Not applicable
Reporting on race, ethnicity, or other socially relevant groupings	In the current study, we use data from human participants that they reported through self-identification, for example their ethnicity and the languages they speak. We do this through informed consent forms, where the study participants fill in how they identify ethnically and what languages they speak.
Population characteristics	Individuals were grouped into populations for population genetic analyses
Recruitment	Local collaborators helped with recruitment of participants
Ethics oversight	Local ethics for sample collection in each country and the Swedish ethics board cleared the analyses and data curation.

Note that full information on the approval of the study protocol must also be provided in the manuscript.

Field-specific reporting

Please select the one below that is the best fit for your research. If you are not sure, read the appropriate sections before making your selection.

☐ Life sciences ☐ Behavioural & social sciences ☒ Ecological, evolutionary & environmental sciences

For a reference copy of the document with all sections, see nature.com/documents/nr-reporting-summary-flat.pdf

Ecological, evolutionary & environmental sciences study design

All studies must disclose on these points even when the disclosure is negative.

Study description	Saliva samples or already extracted DNA samples were obtained from 1308 African individuals from 66 different sites spread over 14 different African countries. DNA was extracted and their mitochondrial genomes were analyzed, in combination with a large database consituted from online available mitochondrial genomes.
Research sample	This study used both newly generated data and publicly available data. The publicly available data was downloaded from NCBI. All newly generated data is from African populations, where we aimed at sampling from diverse regions and cultures, to get a good representation of African populations.
Sampling strategy	We aimed at sampling 20 individuals per population, as this number has been shown to be a good representation of human populations. Only unrelated adult individuals were asked to participate.
Data collection	Several collaborators obrtained the saliva samples from various populations, including written informed consent and metadata.
Timing and spatial scale	Samples were collected in different time periods and different African countries - depending on the specific collaborators.
Data exclusions	We filtered our data with a minimum of 30x coverage.
Reproducibility	There were 16 technical duplicates which gave the same results.
Randomization	Individuals were grouped into populations based on self-reported ethnicity and language
Blinding	Individual DNA is de-identified by using codes. Personal information of individuals are not entered in the study or in online databases

Did the study involve field work? ☒ Yes ☐ No

Field work, collection and transport

Field conditions	Samples were collected from participants who were informed about the study purpose and who signed informed consent forms.
Location	Full information on collection sites are available in the article. Numerous locations across the African continent

Access & import/export	Local ethics clearance were obtained by local collaborators
Disturbance	NA

Reporting for specific materials, systems and methods

We require information from authors about some types of materials, experimental systems and methods used in many studies. Here, indicate whether each material, system or method listed is relevant to your study. If you are not sure if a list item applies to your research, read the appropriate section before selecting a response.

Materials & experimental systems

n/a	Involved in the study
☒	☐ Antibodies
☒	☐ Eukaryotic cell lines
☒	☐ Palaeontology and archaeology
☒	☐ Animals and other organisms
☒	☐ Clinical data
☒	☐ Dual use research of concern
☒	☐ Plants

Methods

n/a	Involved in the study
☒	☐ ChIP-seq
☒	☐ Flow cytometry
☒	☐ MRI-based neuroimaging

Plants

Seed stocks	not applicable
Novel plant genotypes	not applicable
Authentication	not applicable
