# Supplementary material for: Revisiting the African mtDNA landscape through complete mitochondrial genomes
Source: Commun Biol. 2026 Jun 3;9:754. doi: 10.1038/s42003-026-10330-9 (PMC13234189; doi:10.1038/s42003-026-10330-9)
Supplement: Supplementary file 14 — Supplementary Figures and Tables [file 42003_2026_10330_MOESM14_ESM.pdf]

# Supplementary materials

## *Revisiting the African mtDNA Landscape Through Complete Mitochondrial Genomes*

**Supplementary Note 1**
**Supplementary Figures**
**Supplementary Tables**
**Complete reference list**

## Supplementary Note 1: The use of linguistic labels for genetic clusters

In this study, we utilize linguistic labels to refer to genetic clusters among populations. Joseph Greenberg originally proposed four linguistic families (or phyla) for Africa in 1963: [22] Afro-Asiatic, Nilo-Saharan, Niger-Congo, and Khoisan. However, the genealogical validity of some of these phyla has been questioned by several linguists. [24, 23] Among them, Khoisan and Nilo-Saharan are the least accepted. Khoisan languages, initially considered a single family, are currently recognized as three distinct families (Kx'a, Tuu, and Khoe-Kwadi) and two language isolates (Sandawe and Hadza). The use of click consonants and linguistic borrowing led Greenberg to group these languages together as one linguistic family. For the traditional Nilo-Saharan family, constituent families are recognized but the overarching classification as Nilo-Saharan remains contentious. The unity of Niger-Congo languages is also debated, with Mande and Ubangian languages now excluded from this group. The core Niger-Congo families, including Bantu, Bantoid besides Bantu, West-Benue-Congo, Kwa, Kru, Senufu, and Gur, however remain assigned to this phylum. [32] Afro-Asiatic is more widely accepted as a language family, and includes Berber, Chadic, Cushitic, Egyptian, and Semitic languages.

Despite the linguistic obsolescence of the labels Nilo-Saharan, Niger-Congo, and Khoisan, genome-wide genetic analyses by Tishkoff *et al.*, [20] have demonstrated that populations grouped under these four linguistic labels proposed by Greenberg form distinct genetic clusters. Furthermore, the last full overview of mtDNA diversity on the African continent was published by Salas *et al.* and uses the four language phyla proposed by Greenberg. [46] With a need to give an understandable label to the observed genetic clusters, and for continuity with the Salas *et al.* paper, we employ these historical linguistic labels as practical identifiers for genetic studies, acknowledging their genetic validity while recognizing their debated or outdated linguistic basis. This approach allows us to leverage historical classifications that in themselves were based on linguistic, anthropological and geographical deduction to explore meaningful genetic distinctions among populations.

A likely reason for the link between Greenberg's classifications and genetic research is that Greenberg's classification is primarily based on language areas rather than strict genealogical relationships. As a result, Greenberg's approach may give the misleading impression that there is a direct correlation between genes and languages, when in reality, the correlation is more closely tied to geography. This geographic correlation explains why Greenberg's classification might show a stronger association with genetic patterns than more recent linguistic classifications, which focus on the genealogical lineage of languages. It is important to recognize that the discrepancies between genetic data and these newer linguistic classifications do not imply that these classifications are less valid than Greenberg's. As we choose to work with Greenberg's classification to label genetics clusters, it is crucial to explicitly acknowledge that it highlights geographic correlations rather than linguistic genealogical ones, and that we merely use the historical linguistic labels as practical identifiers for genetic studies, due to the lack of purely genetic labels.

## Supplementary Figures

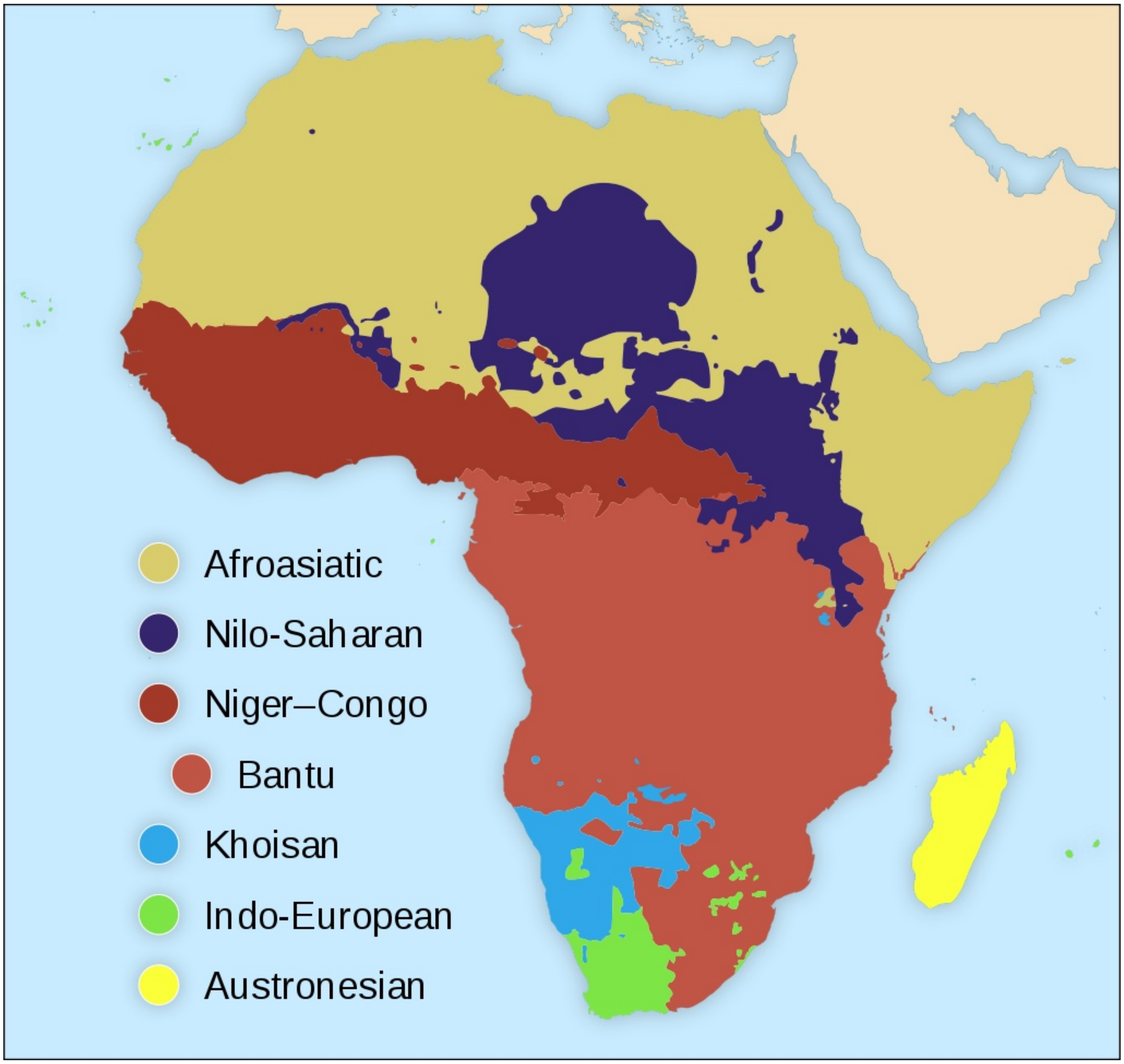


Supplementary Figure 1: Geographical distribution of major language phyla spoken in Africa (taken from Languages of Africa, licensed under CC BY-SA 4.0.). Figure corresponds to Figure 4.1 of The Oxford Handbook of African Archaeology [102] and was proposed by Greenberg in 1963. [22] The language group assignment in this study is not based on the geographical information shown here; rather, it was done with careful assessment of languages spoken by the individuals, based on associated literature. These figures are meant to be used as a general overview of the distribution of language groups and language families. The language classification "Austronesian" as provided in A is not used throughout this paper.

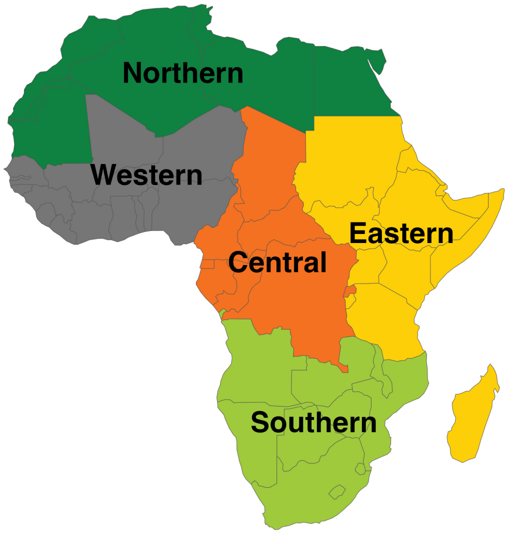


Supplementary Figure 2: Classification of geographical areas into Northern, Western, Central, Eastern and Southern Africa.

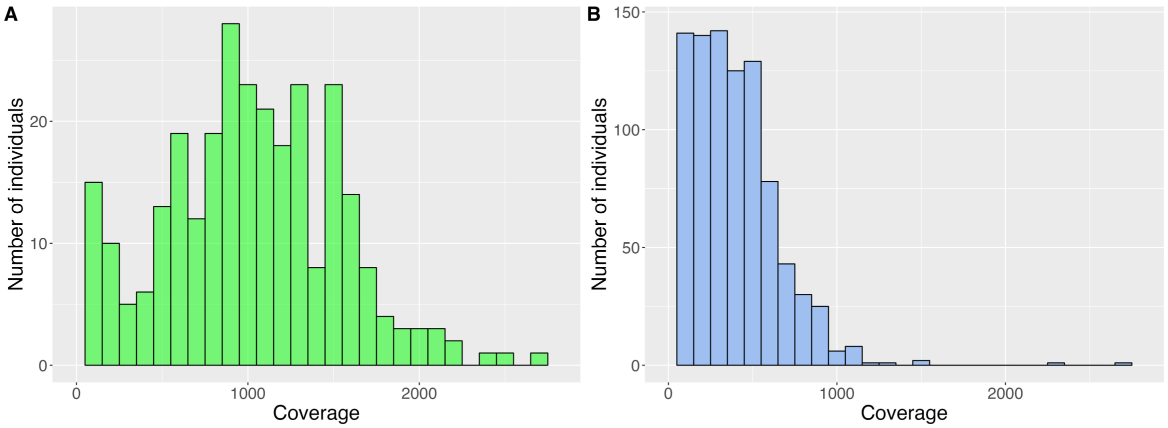


Supplementary Figure 3: Histogram of mtDNA coverage for A) sequencing run 1, in which 292 samples were included. Average coverage is 994x, and B) for sequencing run 2, in which 1,024 samples were included. Average coverage is 340x.

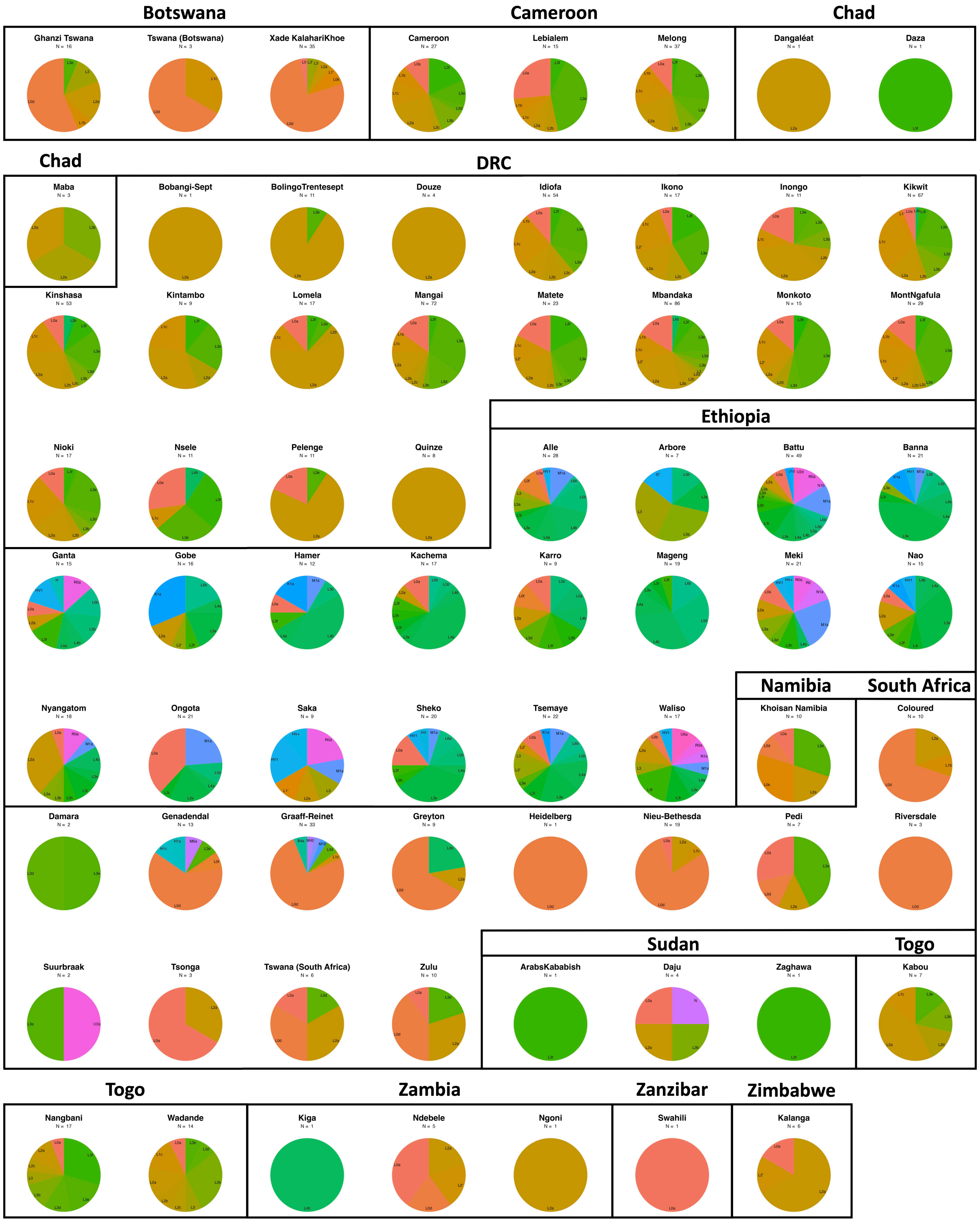


Supplementary Figure 4: The mitochondrial haplogroups found among the studied individuals from the 71 sampling sites. Haplogroups were reduced to three-digit haplogroups. The number of individuals at each site is specified. Sites are grouped based on country. Colours do not correspond to ancestries reported in Supplementary Figure 5.

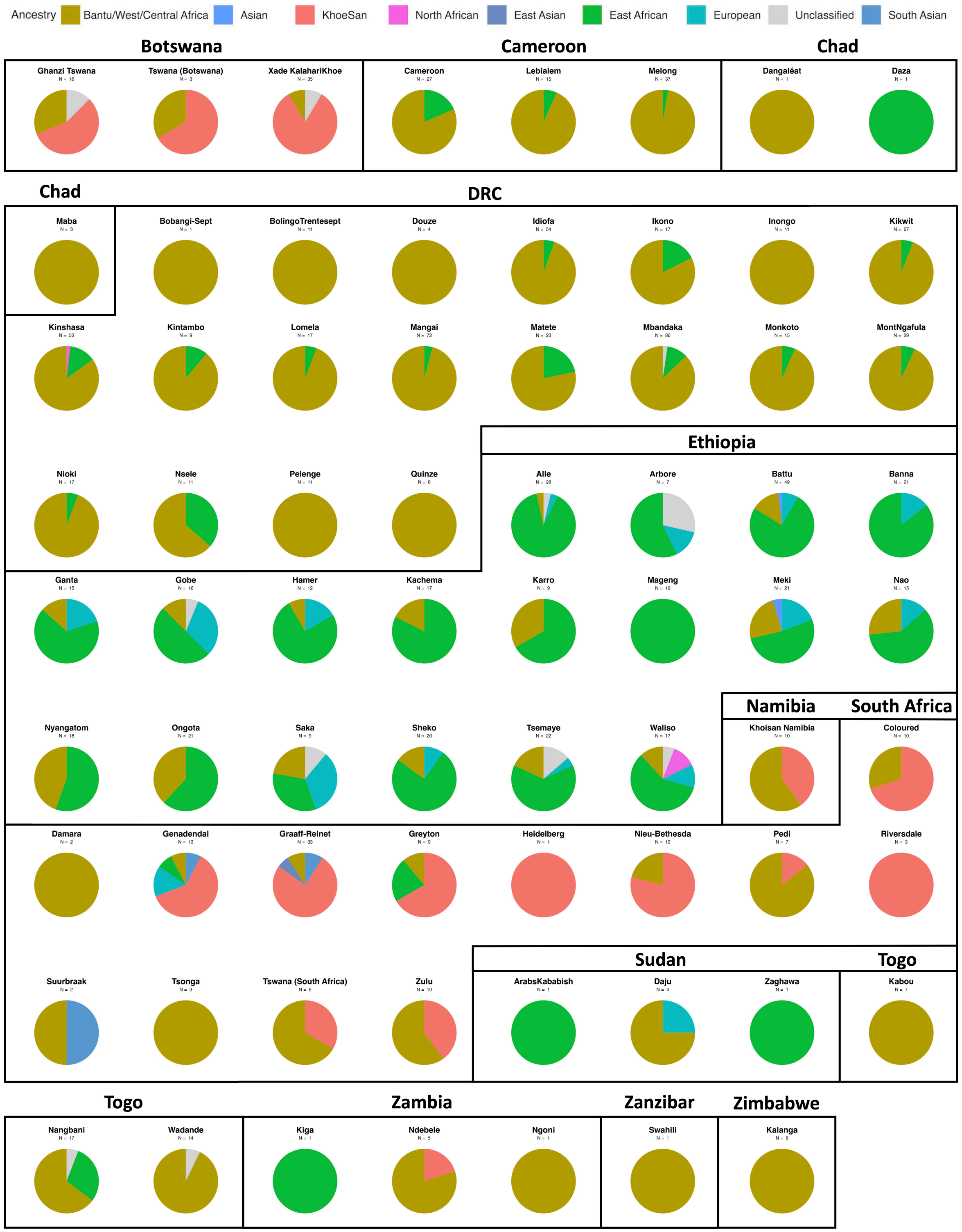


Supplementary Figure 5: Maternal ancestries of individuals from 71 different sampling sites across 13 countries were determined by referencing maternal haplogroups in the literature (see Supplementary Table 1). The number of individuals at each site is specified. Sites are grouped based on country.

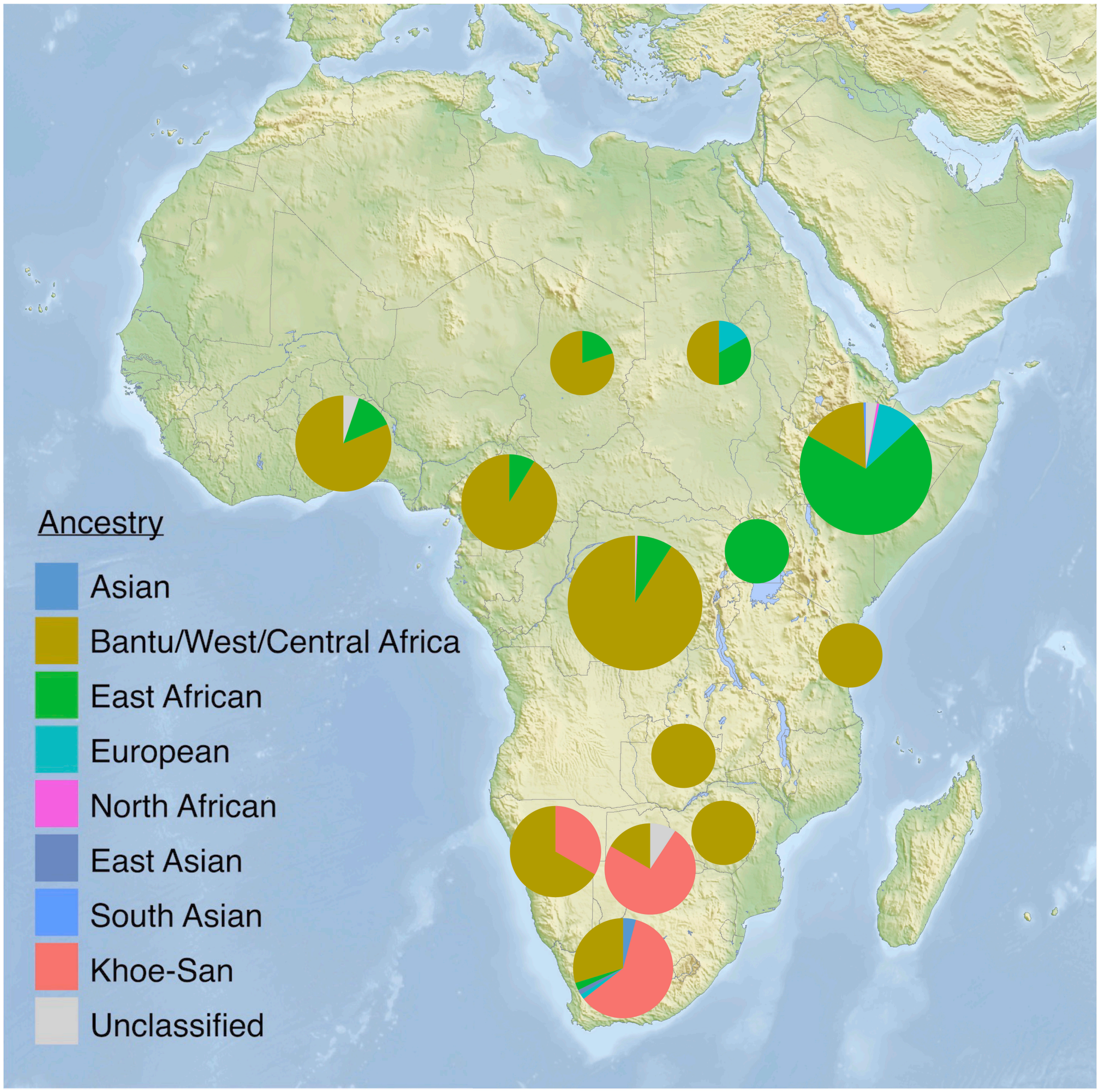


Supplementary Figure 6: Maternal ancestries of individuals per country, visualized on a map. Ancestries were determined by referencing maternal haplogroups in the literature (see Supplementary Table 1).

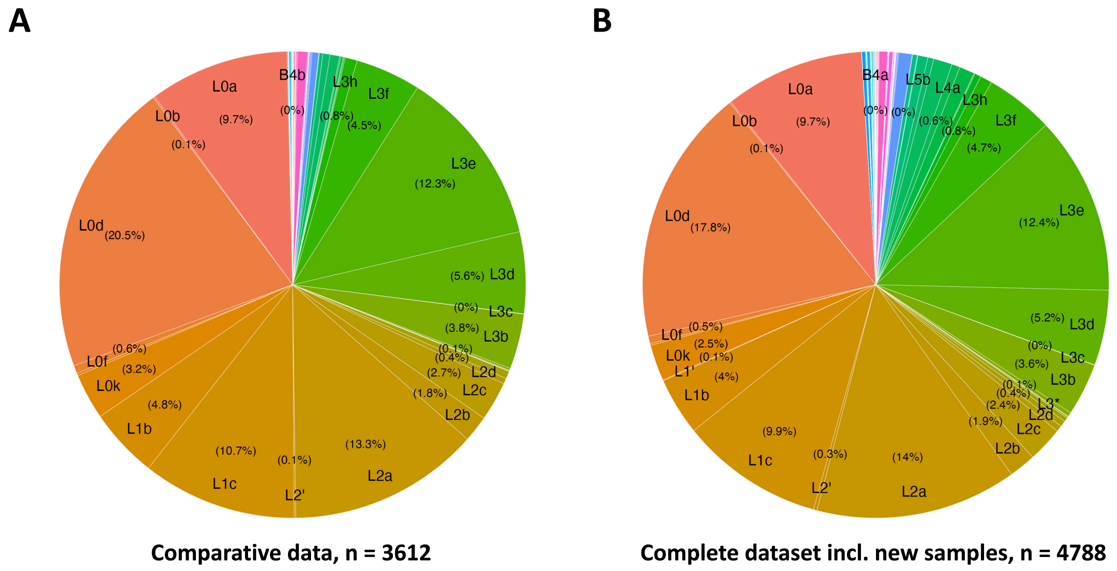


A

Supplementary Figure 7: Distribution of the mitochondrial haplogroups of A) the 3,612 individuals in the comparative dataset, and B) the 4,788 individuals in the complete dataset. This includes the newly sequenced 1,176 individuals. The distribution of mitochondrial haplogroups shown here is heavily influenced by sampling bias (a focus on Khoe-San groups in published literature can probably explain why L0d is the most frequent mitochondrial haplogroup in our dataset). This is therefore not necessarily a reflection of the "real" frequency of mitochondrial haplogroups across the African continent.

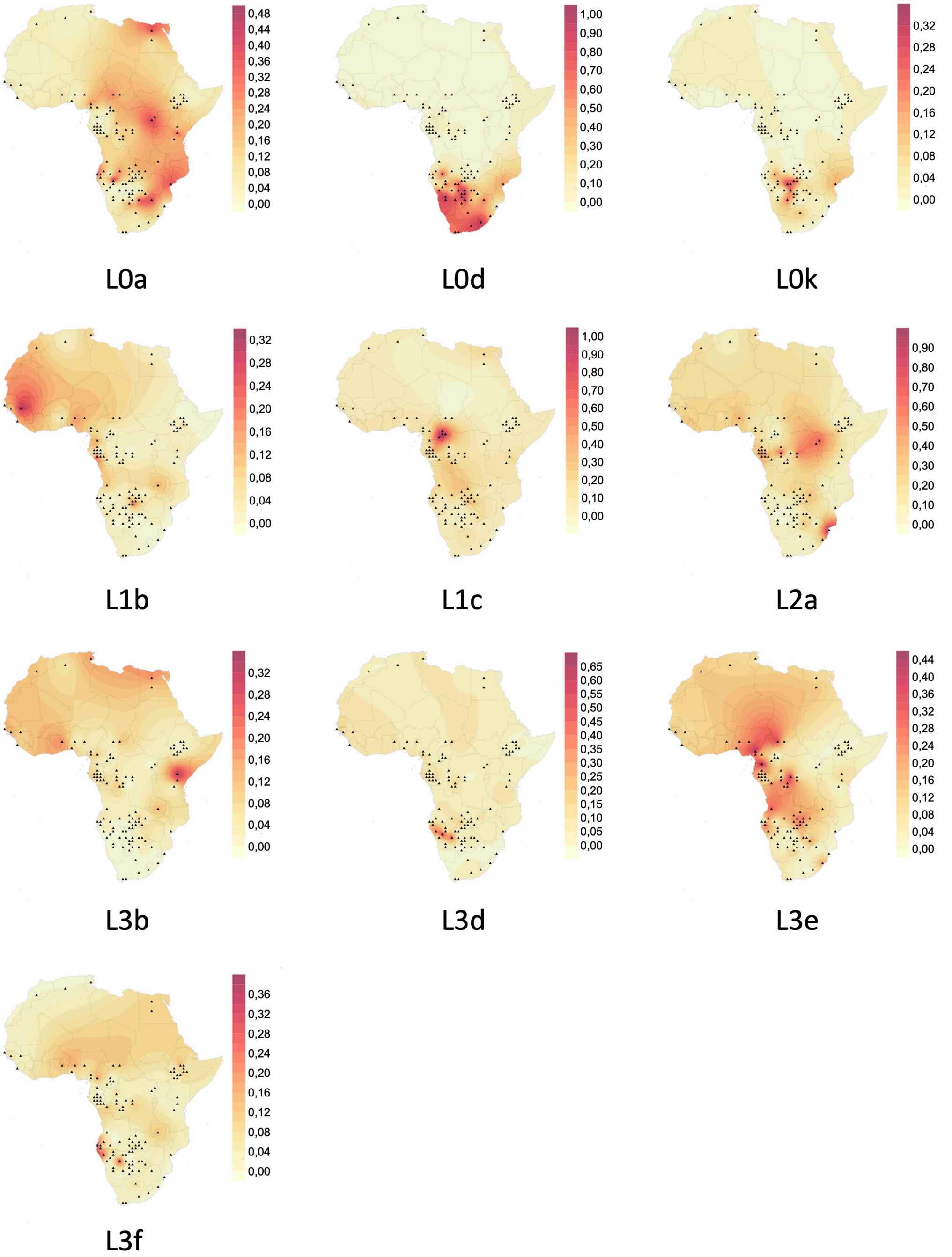


Supplementary Figure 8: Surfer maps of spatial distribution of the haplogroup frequencies for the 10 haplogroups most commonly found in our dataset. The Kriging method was applied. Note that the intensities of the colours are not comparable between the different subfigures.

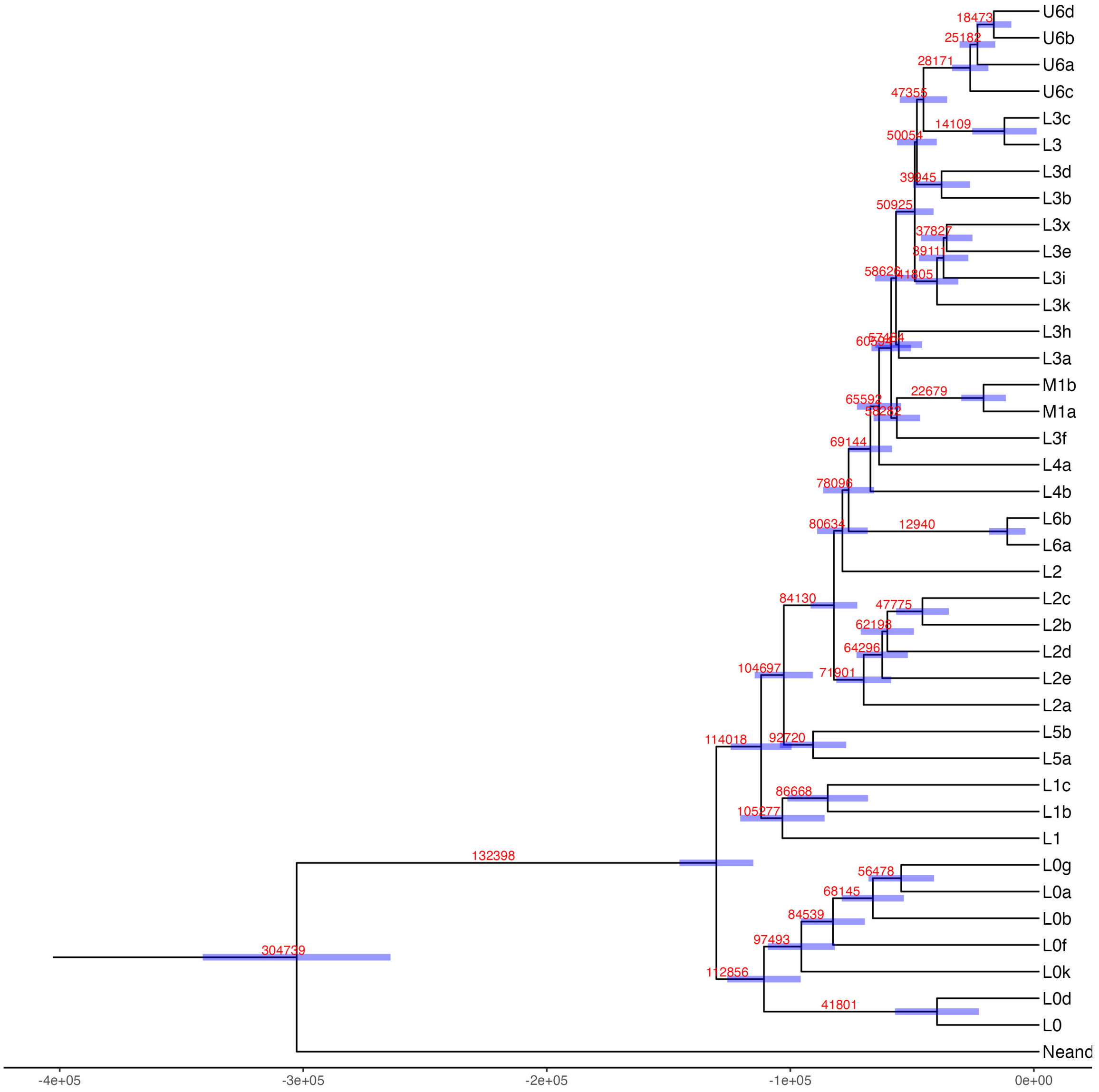


Supplementary Figure 9: The Bayesian tree topology of African mitochondrial haplogroups with Time to Most Recent Common Ancestor (TMRCA) information. Blue bars indicate the 95% confidence intervals of common ancestor heights; in red the mean common ancestor heights are shown.

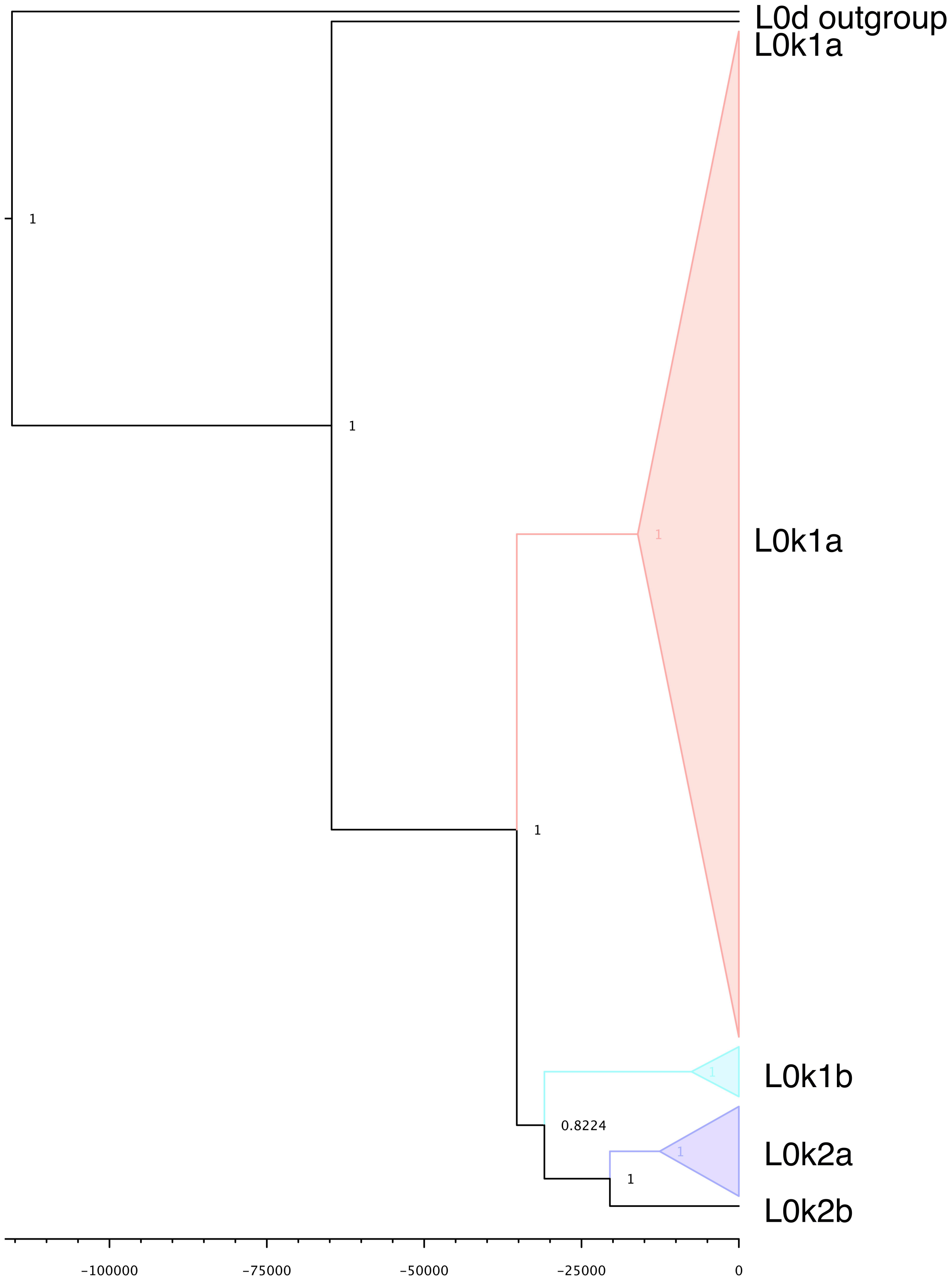


Supplementary Figure 10: A maximum clade credibility tree for all the sequences in the dataset belonging to mitochondrial haplogroup L0k. Node heights represent common ancestor heights. Posterior probability is given at the nodes. On the X-axis, the years in the past are denoted.

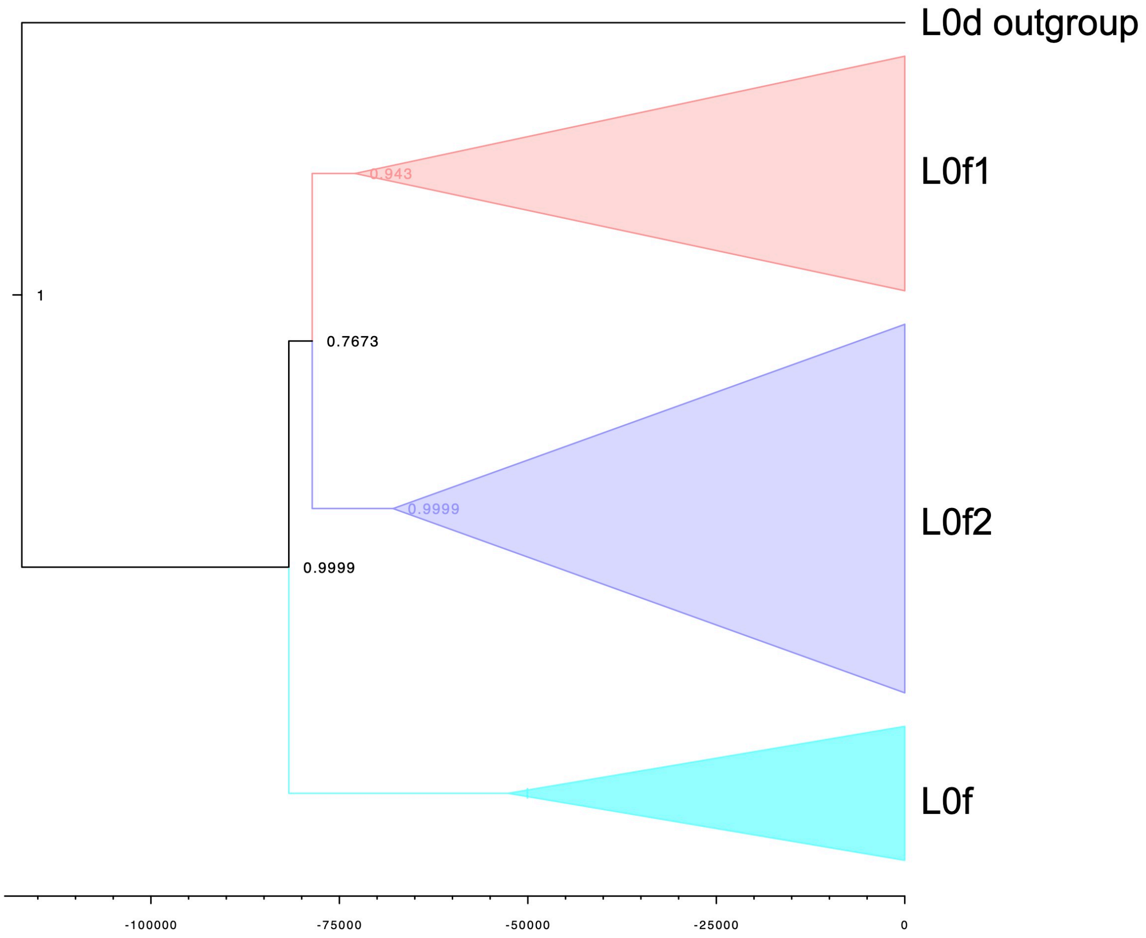


Supplementary Figure 11: A maximum clade credibility tree for all the sequences in the dataset belonging to mitochondrial haplogroup L0f. Node heights represent common ancestor heights. Posterior probability is given at the nodes. On the X-axis, the years in the past are denoted.

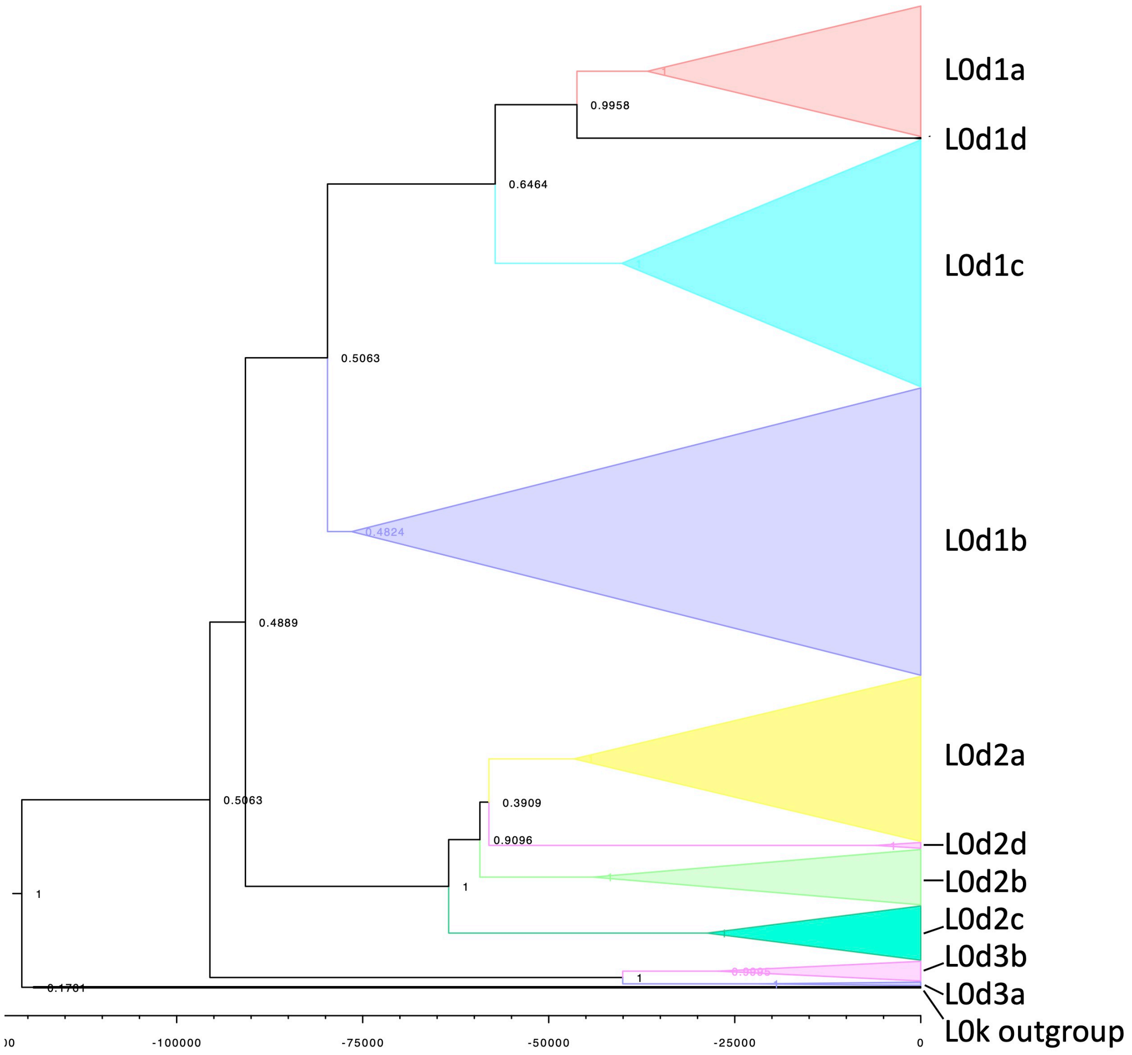


Supplementary Figure 12: A maximum clade credibility tree for all the sequences in the dataset belonging to mitochondrial haplogroup L0d. Node heights represent common ancestor heights. Posterior probability is given at the nodes. On the X-axis, the years in the past are denoted.

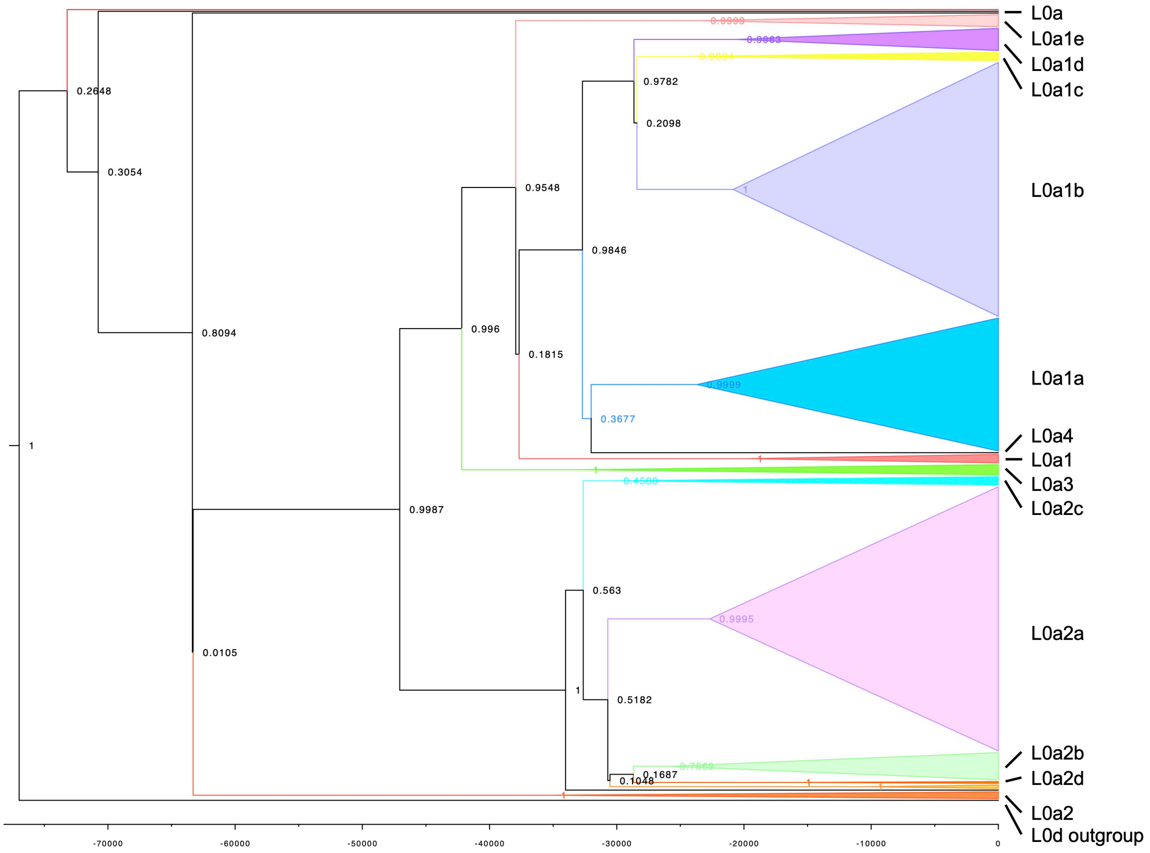


Supplementary Figure 13: A maximum clade credibility tree for all the sequences in the dataset belonging to mitochondrial haplogroup L0a. Node heights represent common ancestor heights. Posterior probability is given at the nodes. On the X-axis, the years in the past are denoted.

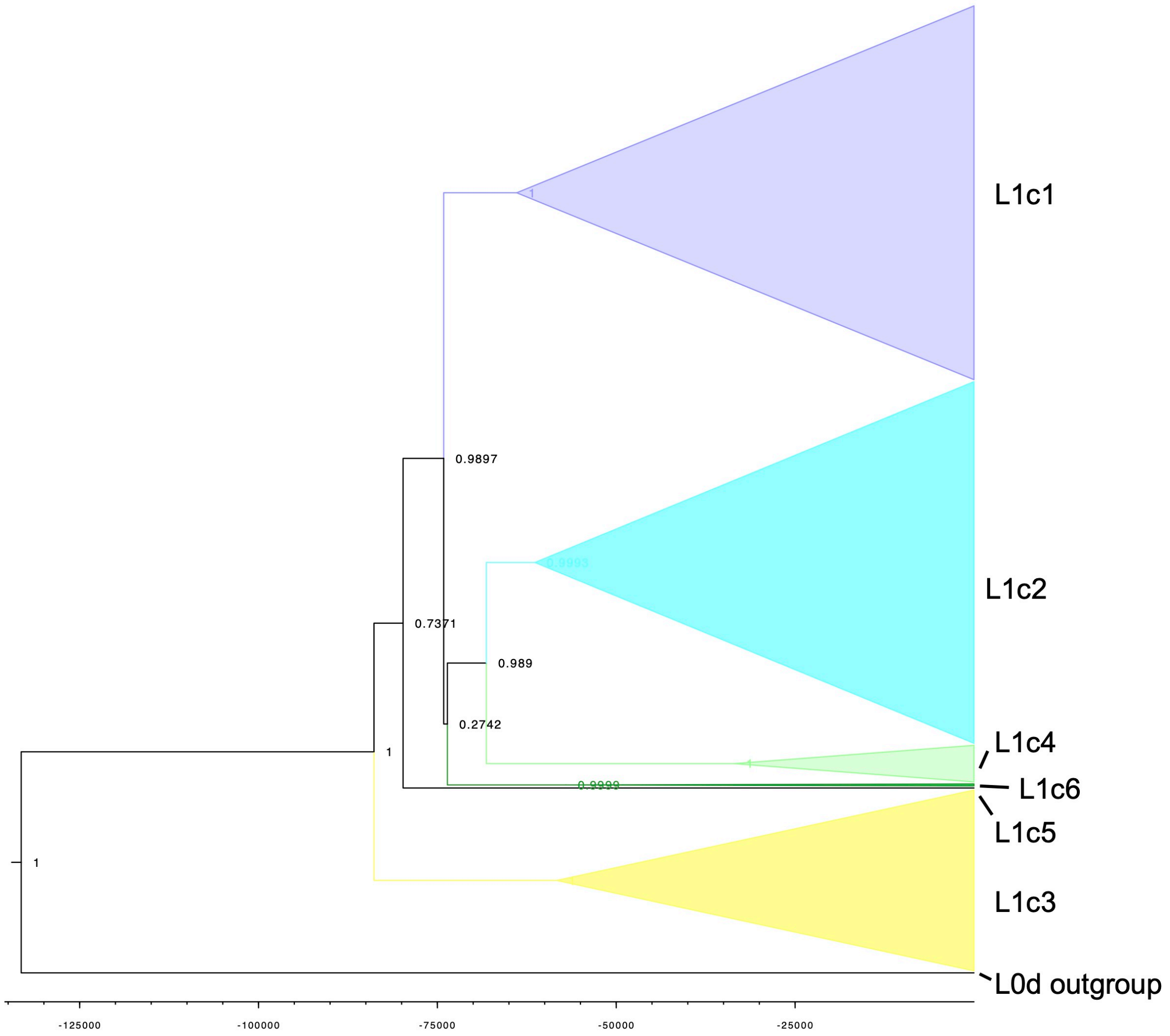


Supplementary Figure 14: A maximum clade credibility tree for all the sequences in the dataset belonging to mitochondrial haplogroup L1c. Node heights represent common ancestor heights. Posterior probability is given at the nodes. On the X-axis, the years in the past are denoted.

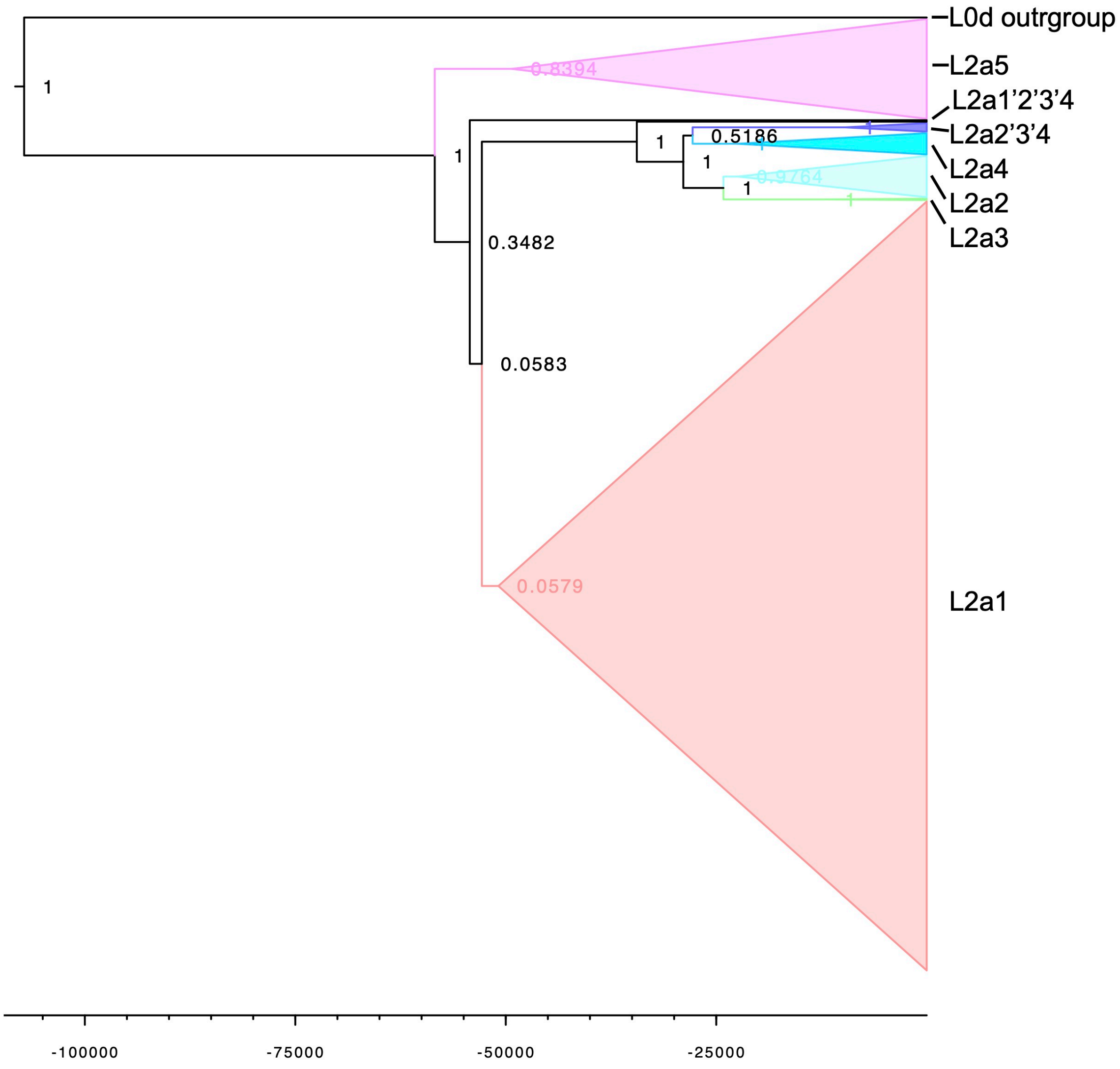


Supplementary Figure 15: A maximum clade credibility tree for all the sequences in the dataset belonging to mitochondrial haplogroup L2a. Node heights represent common ancestor heights. Posterior probability is given at the nodes. On the X-axis, the years in the past are denoted.

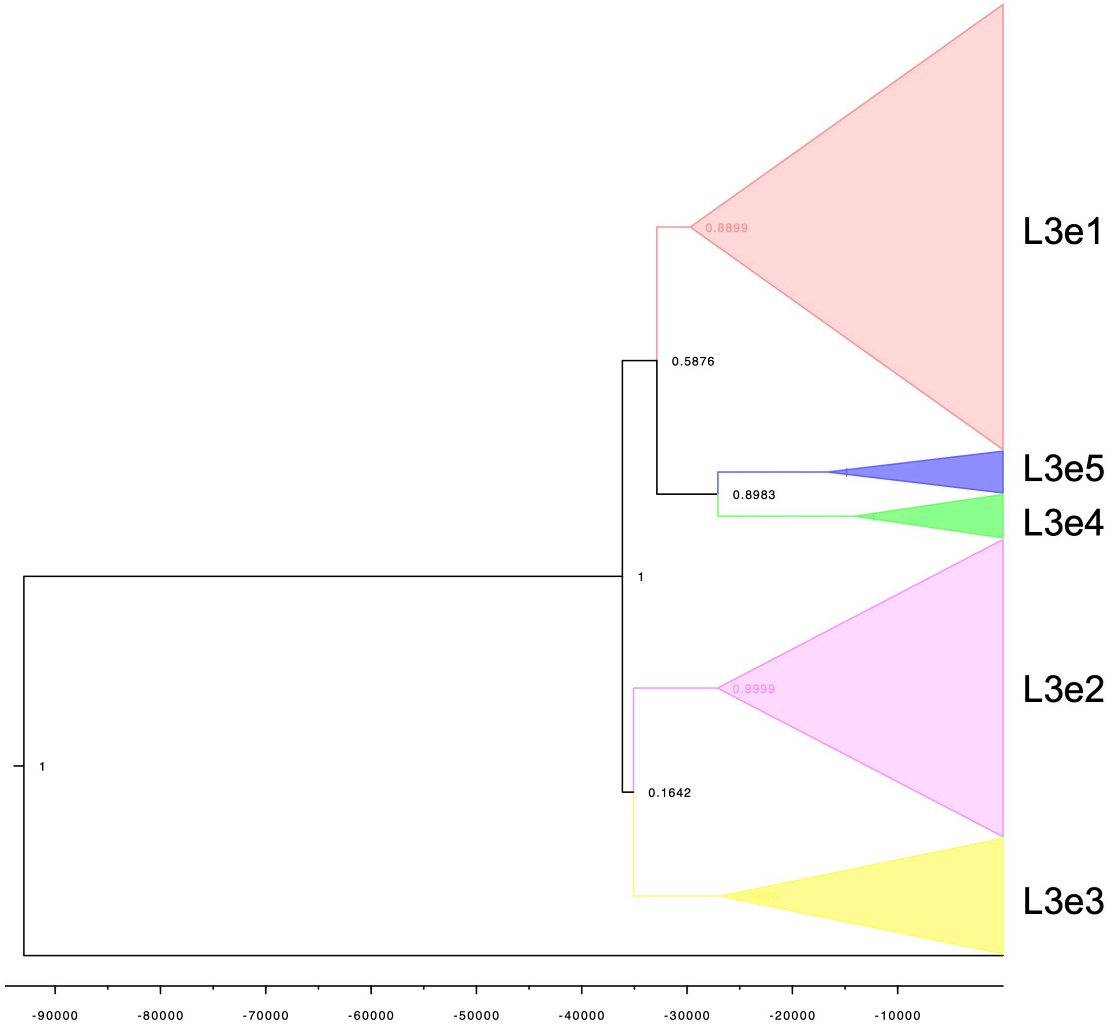


Supplementary Figure 16: A maximum clade credibility tree for all the sequences in the dataset belonging to mitochondrial haplogroup L3e. Node heights represent common ancestor heights. Posterior probability is given at the nodes. On the X-axis, the years in the past are denoted.

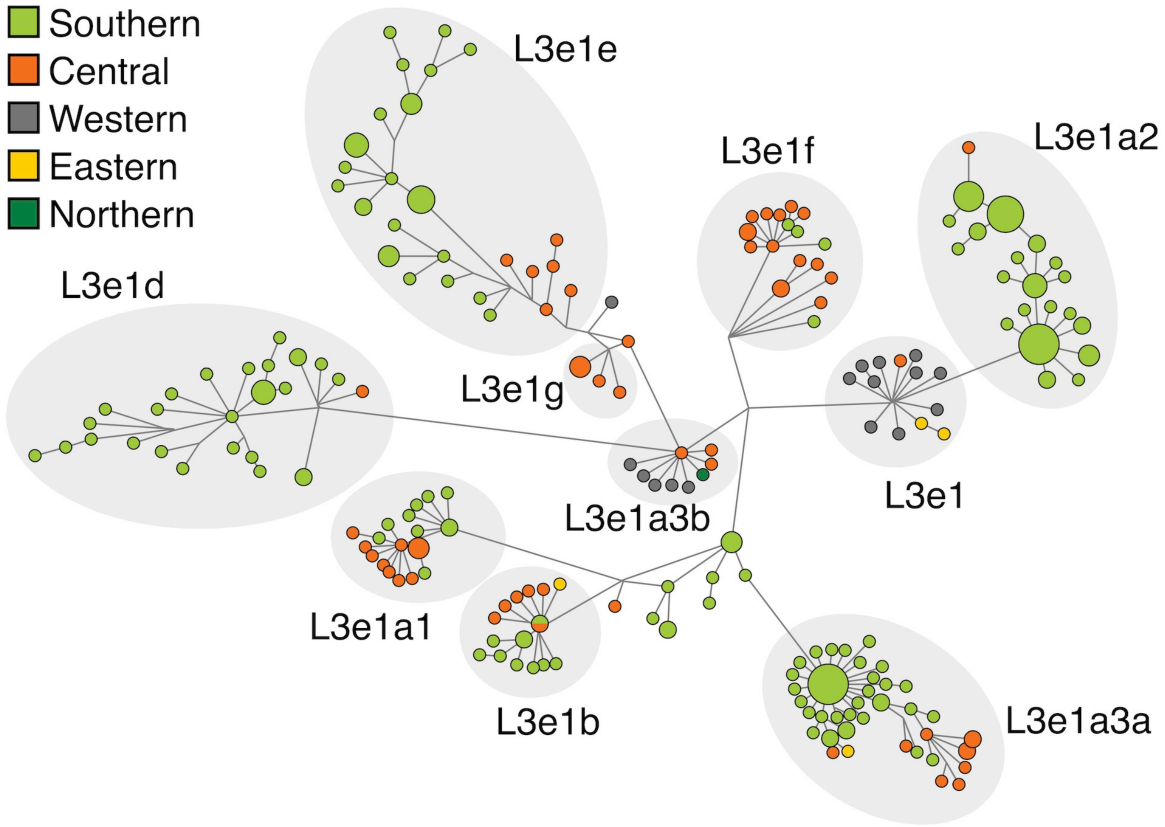


Supplementary Figure 17: Median joining network of all sequences belonging to mitochondrial haplogroup L3e1.

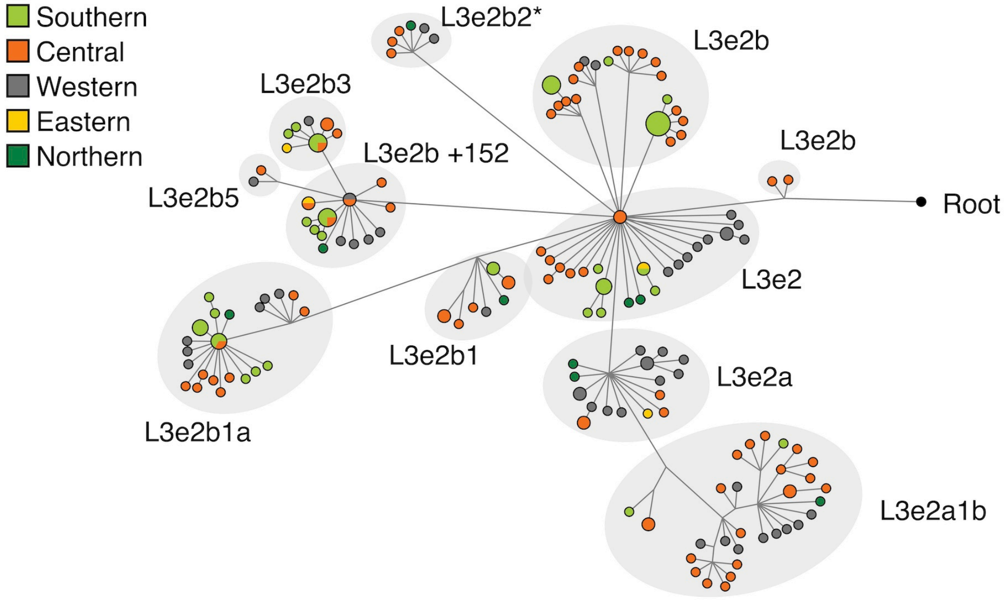


Supplementary Figure 18: Median joining network of all sequences belonging to mitochondrial haplogroup L3e2.

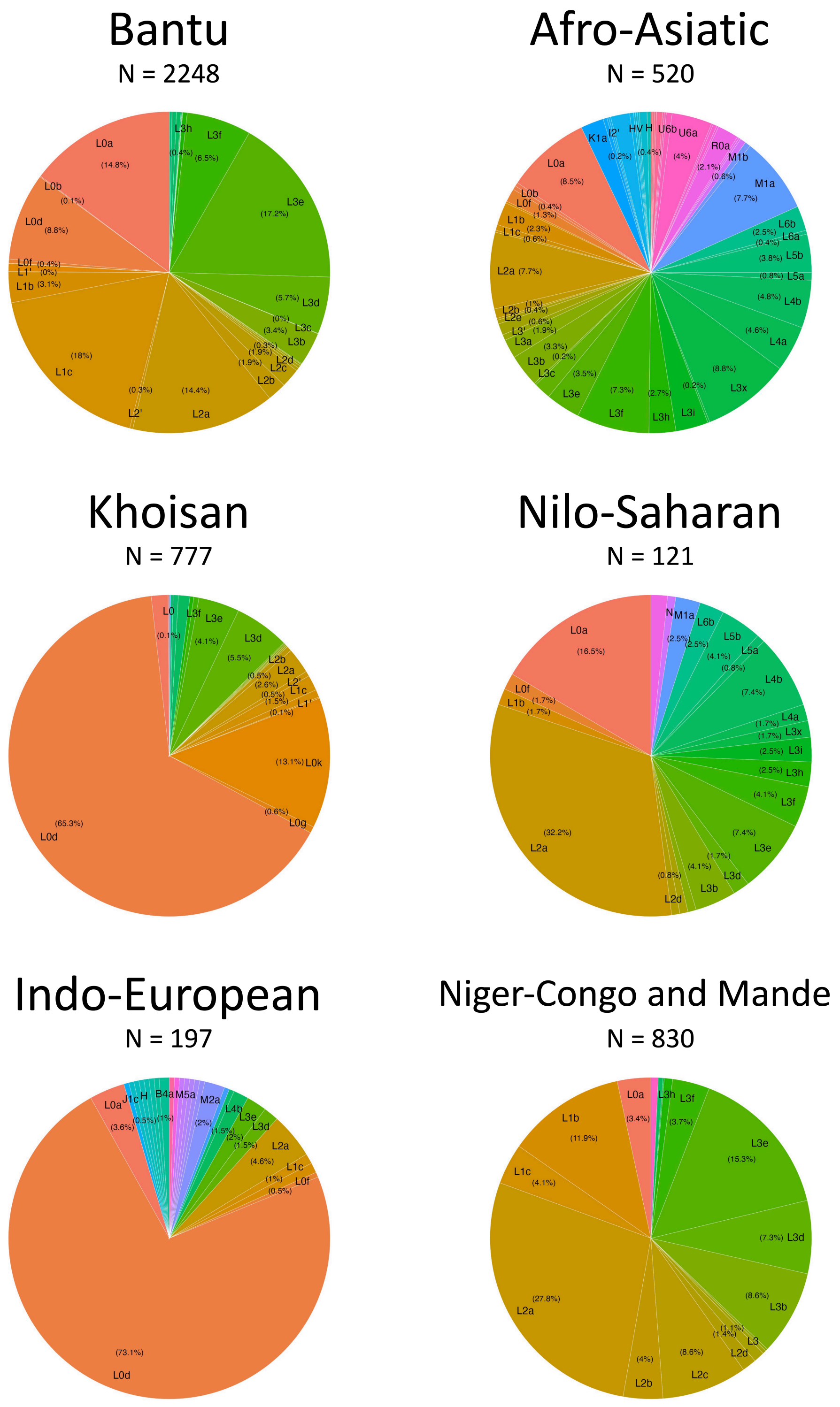


Supplementary Figure 19: Distribution of the mitochondrial haplogroups among the different language groups.

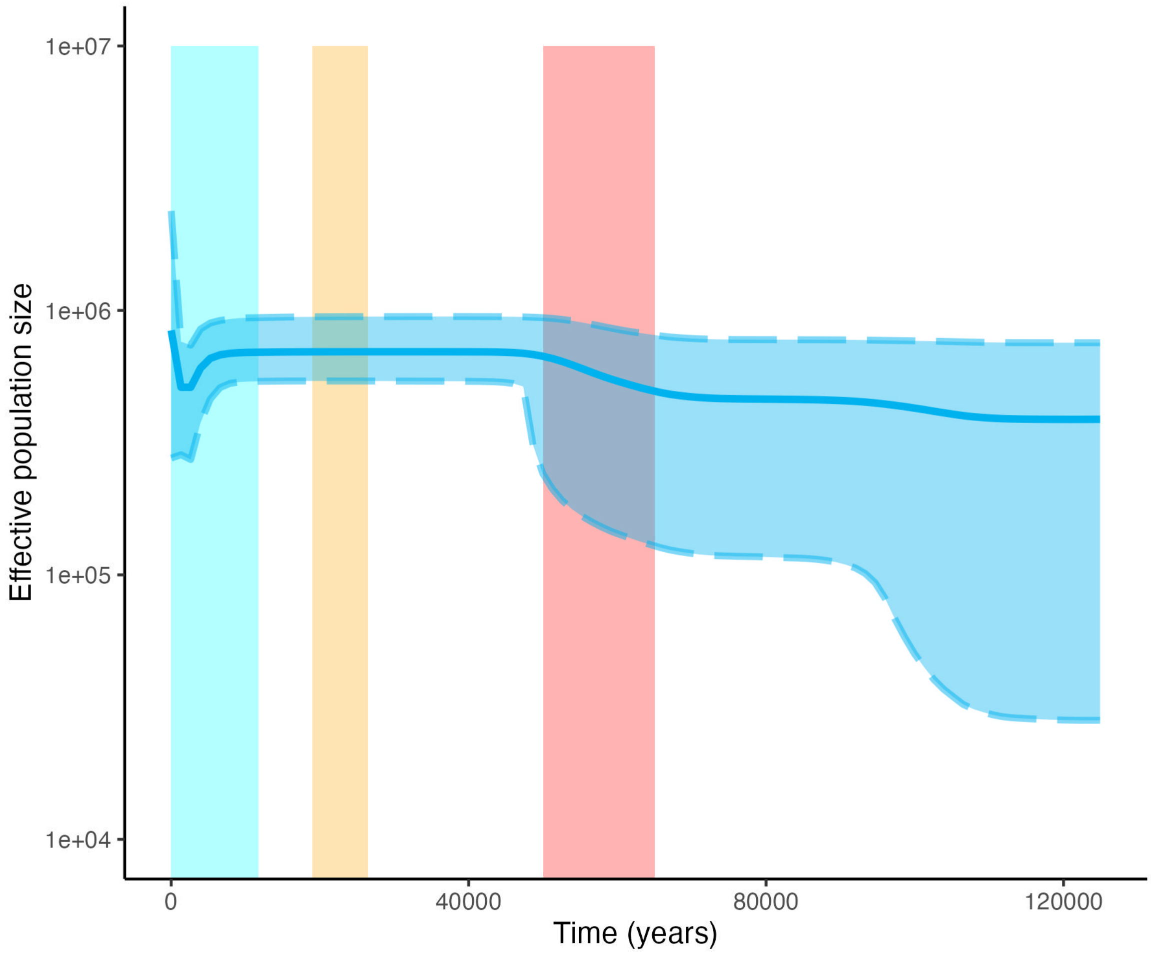


Supplementary Figure 20: Bayesian Skyline Plots showing the variation of female $N_e$ through time for all Khoisan speakers. The bold middle line represents the mean estimates and the two thinner lines surrounding the bold line represent the 95% highest posterior density (HPD) intervals. The red area denotes the time of the out-of-Africa migration (65–50 kya), the yellow area the Last Glacial Maximum (LGM)(26.5–19 kya), and the blue area the Holocene (last 11.7 ky). For the generation of this plot, 777 individuals were included.

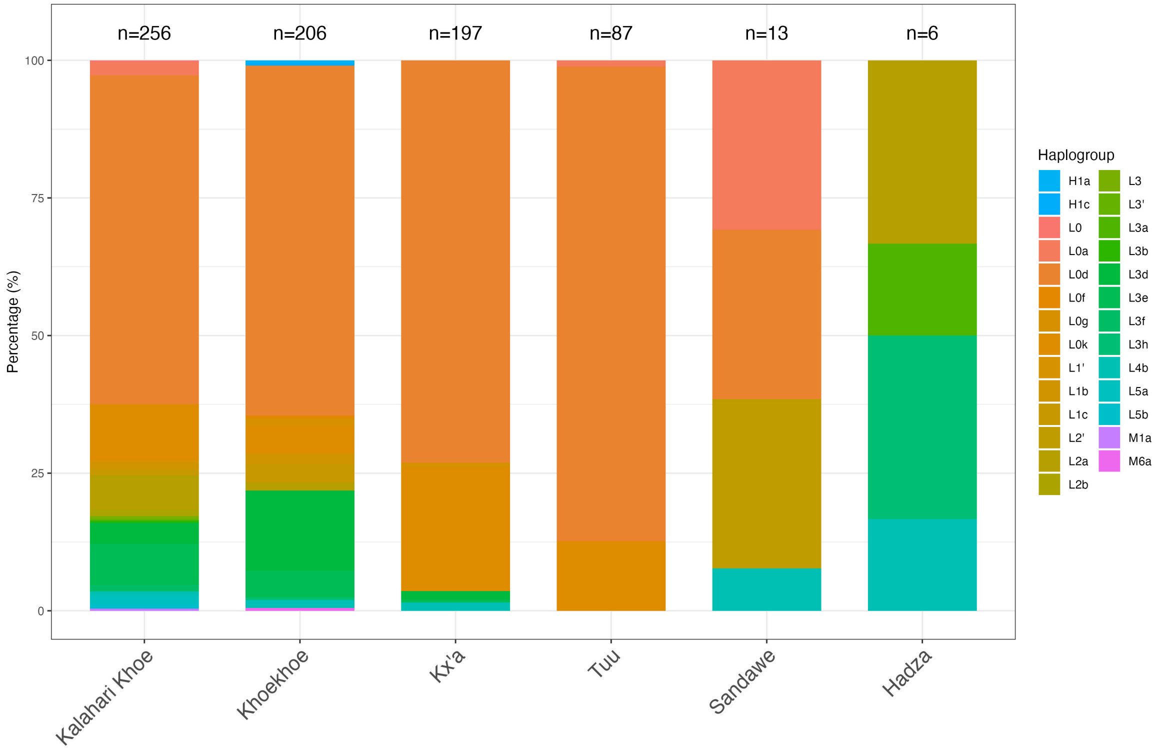


Supplementary Figure 21: Haplogroup distribution among the six Khoisan languages. Number of individuals in each group is indicated at the top of each bar.

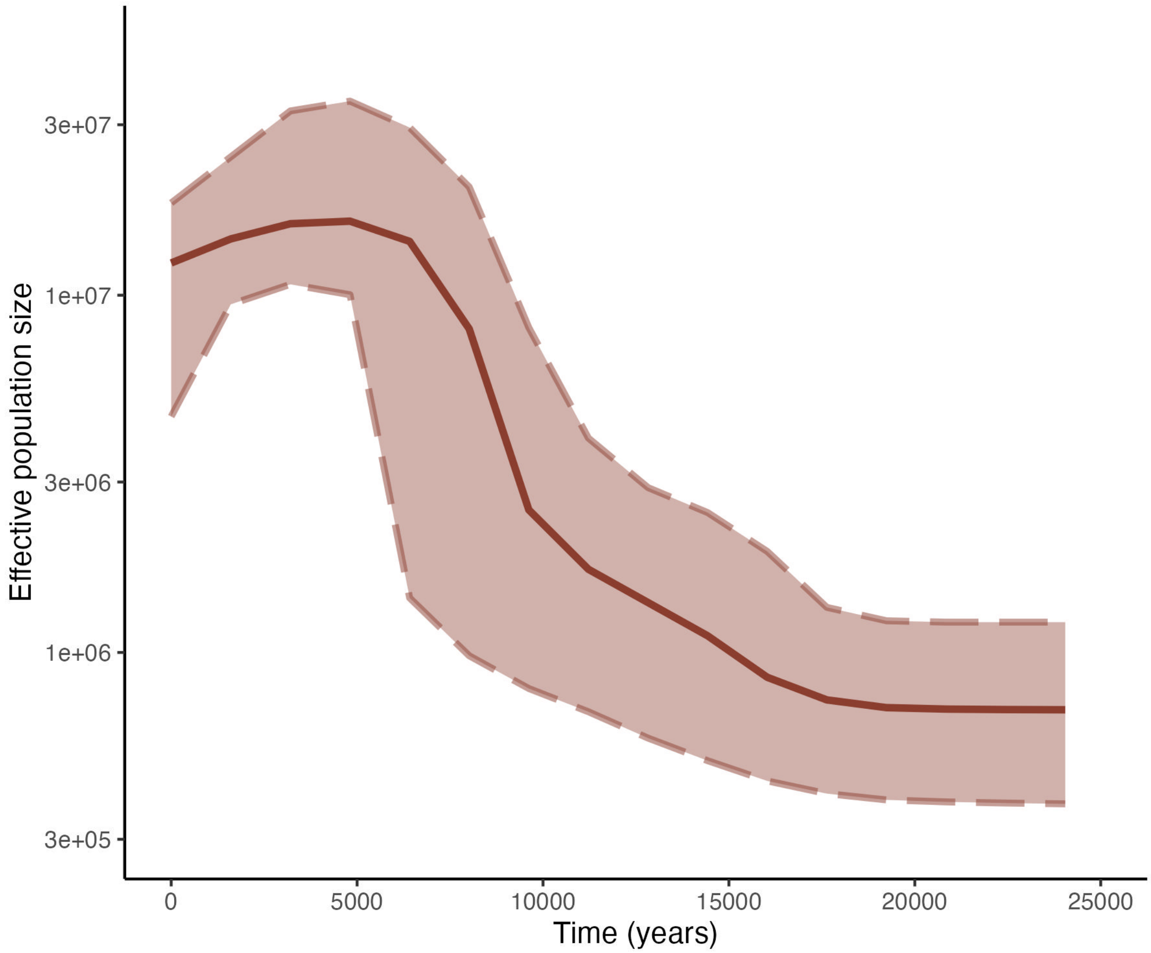


Supplementary Figure 22: Bayesian Skyline Plots showing the variation of $N_e$ through time for Niger-Congo and Mande speakers, excluding Bantu speakers. For the generation of this plot, 830 individuals were included.

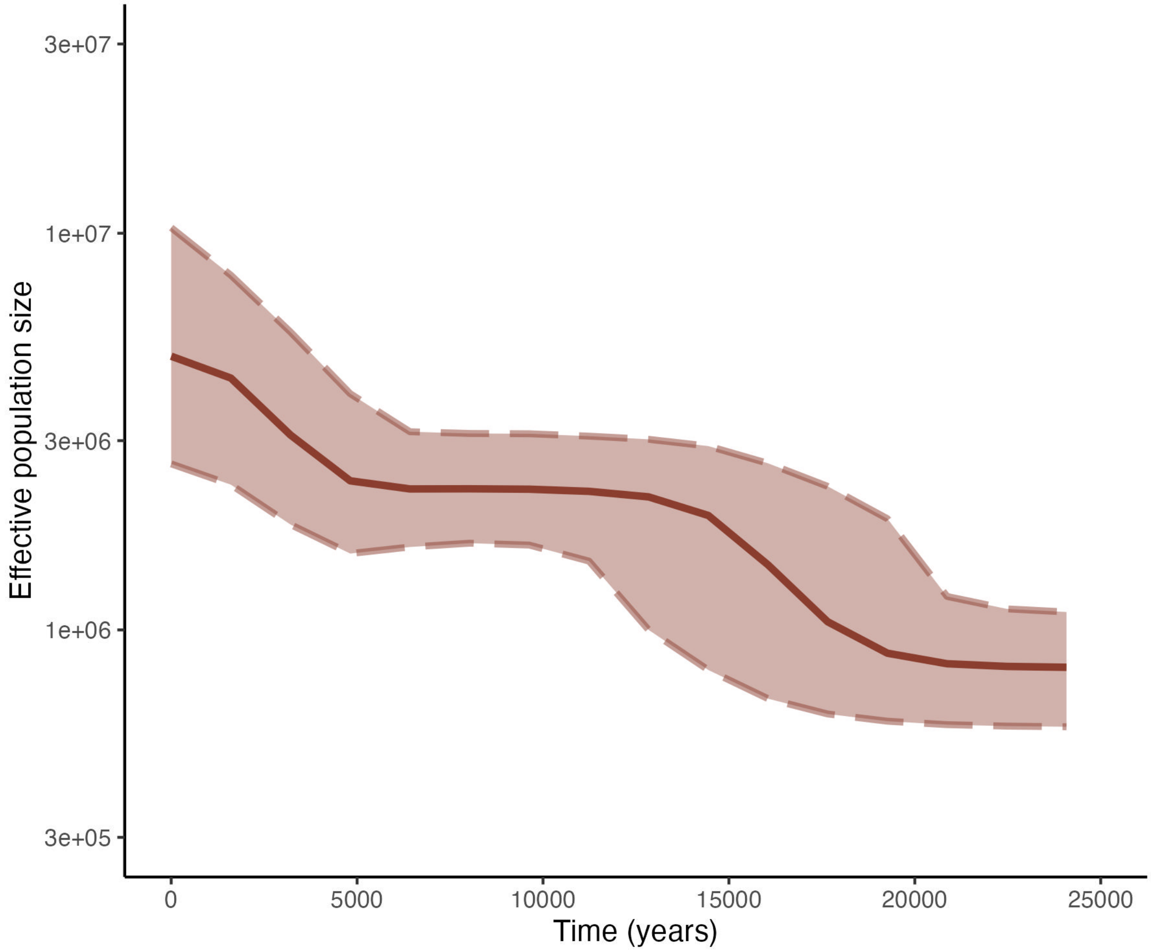


Supplementary Figure 23: Bayesian Skyline Plots showing the variation of $N_e$ through time for Niger-Congo and Mande speakers, including Bantu speakers. For the generation of this plot, a subset of all Niger-Congo + Mande and Bantu speakers was taken. The group contained 500 individuals; 365 Bantu speakers and 135 Niger Congo + Mande speakers.

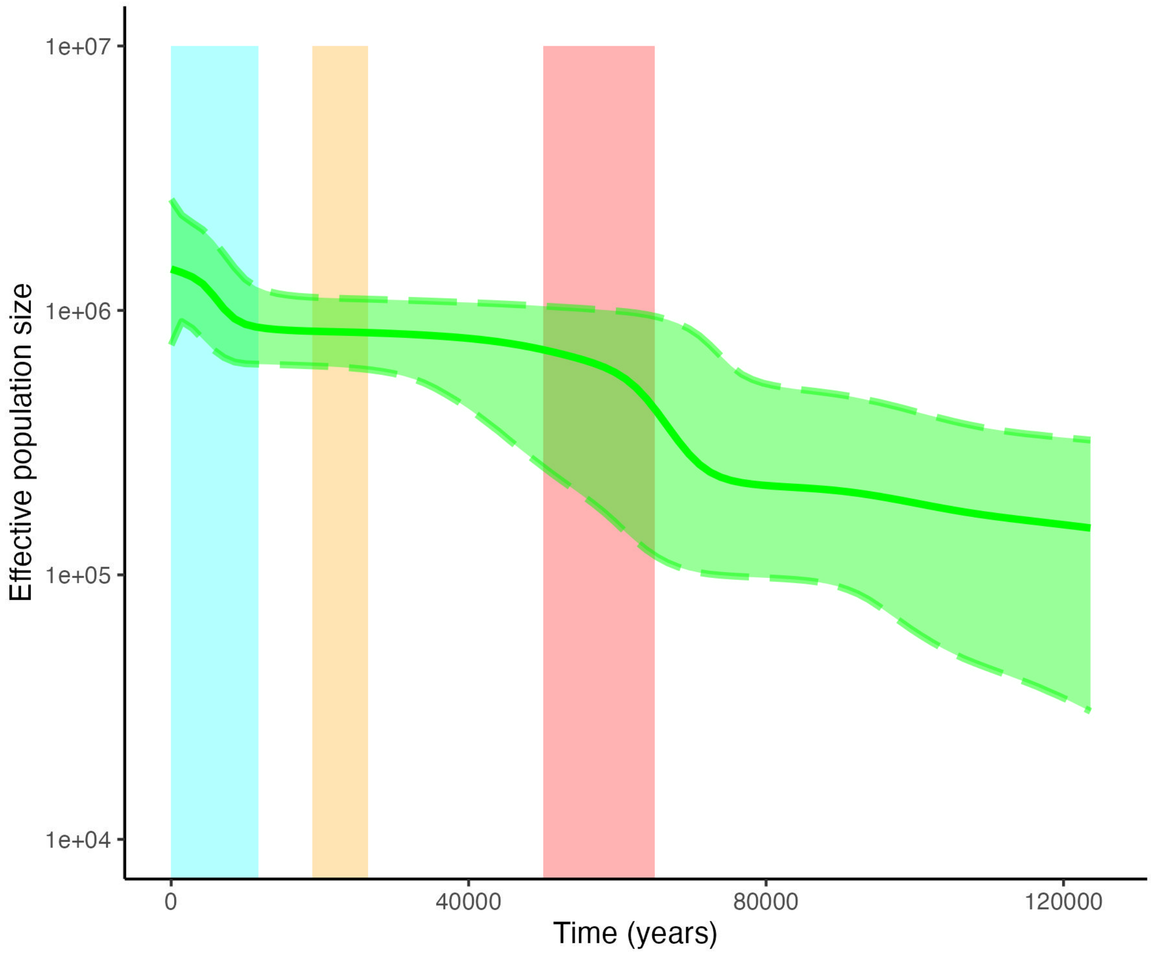


Supplementary Figure 24: Bayesian Skyline Plots showing the variation of $N_e$ through time for all Afrikaans speakers in the dataset. The bold middle line represents the mean estimates and the two thinner lines surrounding the bold line represent the 95% highest posterior density (HPD) intervals. The red area denotes the time of the out-of-Africa migration (65–50 kya), the yellow area the Last Glacial Maximum (LGM)(26.5–19 kya), and the blue the Holocene (last 11.7 ky). For the generation of this plot, 197 individuals were included.

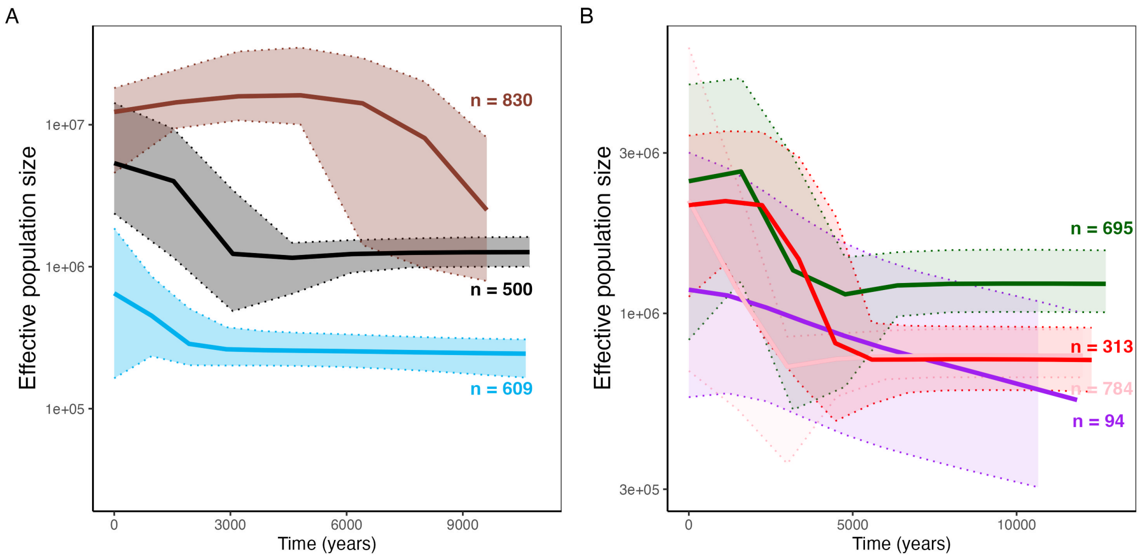


Supplementary Figure 25: Bayesian Skyline Plots showing the variation of $N_e$ through time for various groups. In panel A) the BSPs of Niger-Congo and Mande speakers (brown), Bantu speakers (black) and Khoisan speakers (blue) are shown, including the confidence intervals. In panel B) the BSPs of the North-Western Bantu (purple), West-Western Bantu (red), South-Western Bantu (pink) and Eastern Bantu (green) are shown, including the confidence intervals. The number of individuals used for each BSP is indicated next to the line in the corresponding colour.

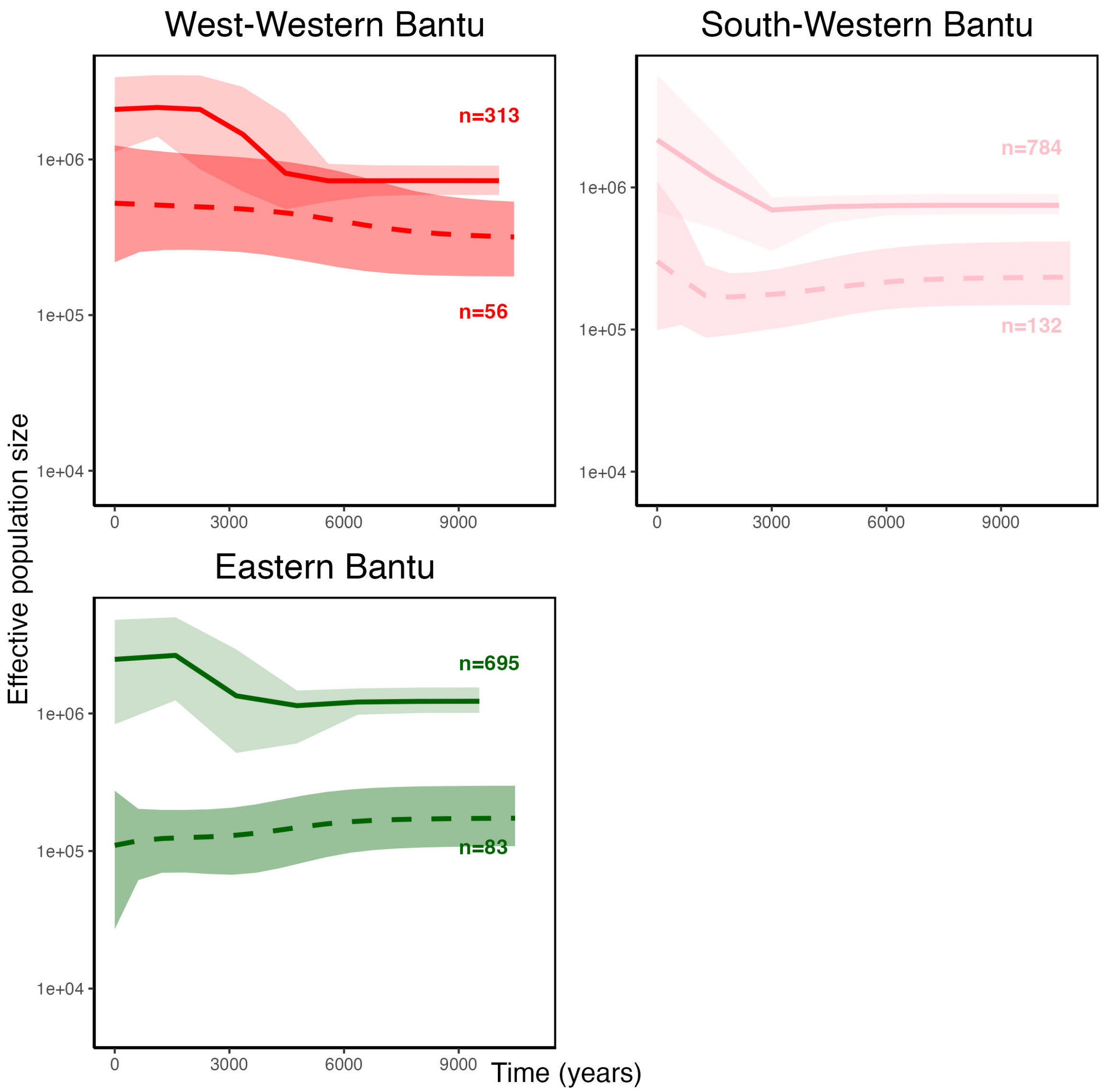


Supplementary Figure 26: Bayesian Skyline Plots showing the variation of $N_e$ through time estimated from L1c carriers of separate Bantu speaker groups. The number of individuals used for each BSP is indicated next to the line in the corresponding colour. The continuous lines represent the BSP of all speakers of the corresponding language, the dashed lines represent the BSP of speakers of the corresponding language carrying haplogroup L1c.

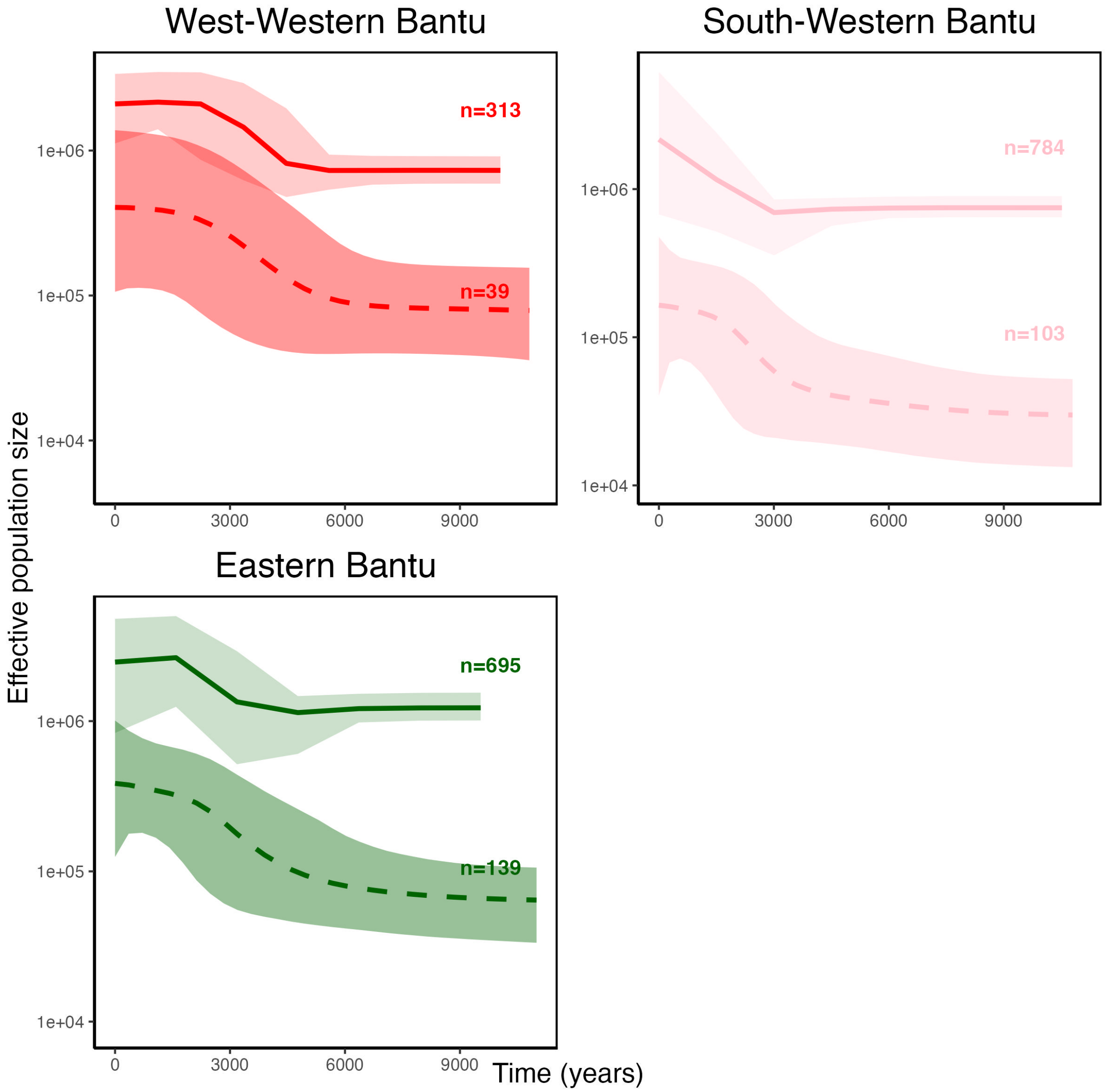


Supplementary Figure 27: Bayesian Skyline Plots showing the variation of $N_e$ through time estimated from L0a carriers of separate Bantu speaker groups. Bayesian Skyline analysis for L0a carriers in North-Western Bantu speakers was not possible due to the low number of individuals with this haplogroup among them. The number of individuals used for each BSP is indicated next to the line in the corresponding colour. The continuous lines represent the BSP of all speakers of the corresponding language, the dashed lines represent the BSP of speakers of the corresponding language carrying haplogroup L0a.

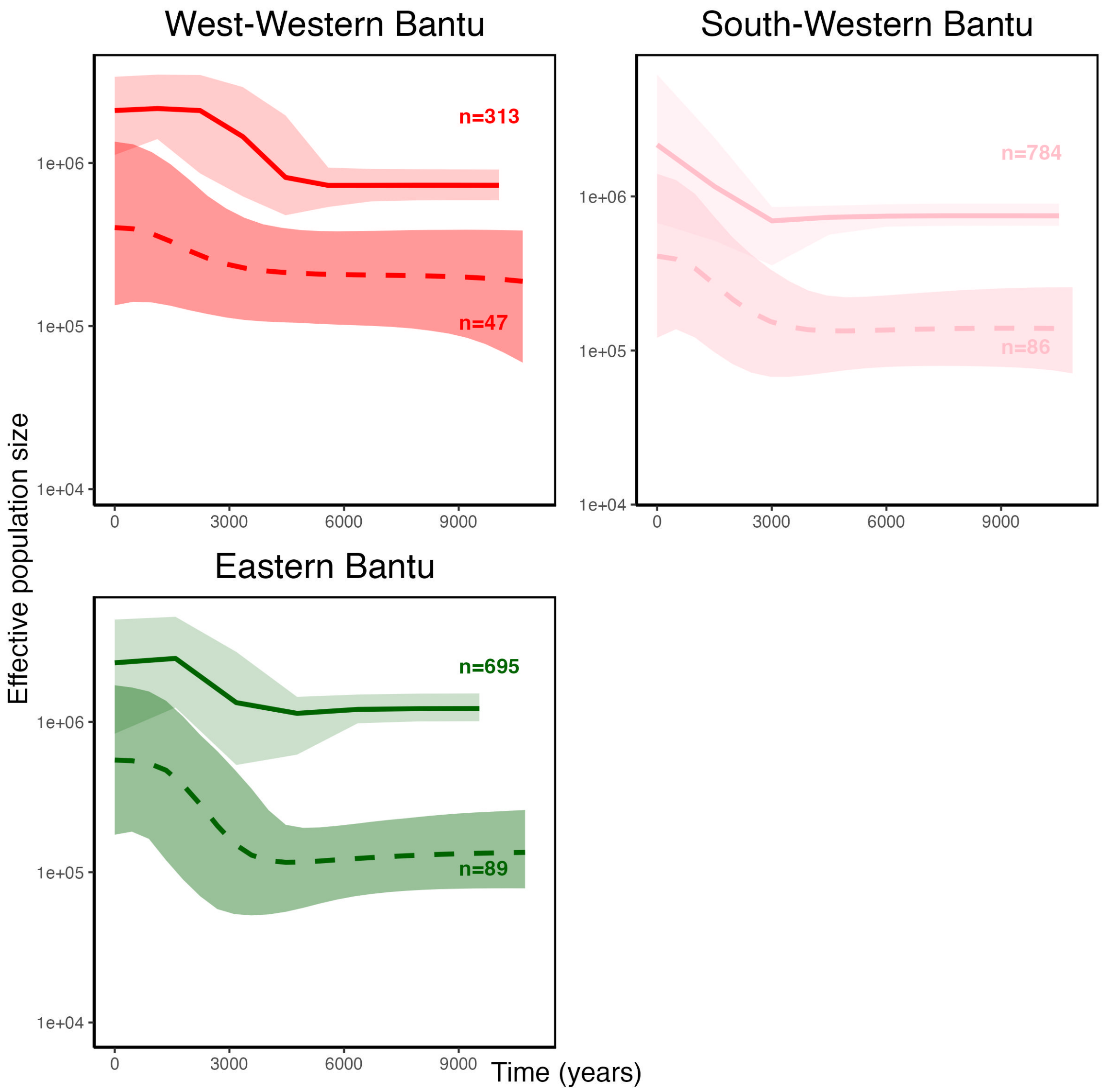


Supplementary Figure 28: Bayesian Skyline Plots showing the variation of $N_e$ through time estimated from L2a carriers of separate Bantu speaker groups. Bayesian Skyline analysis for L2a carriers in North-Western Bantu speakers was not possible due to the low number of individuals with this haplogroup among them. The number of individuals used for each BSP is indicated next to the line in the corresponding colour. The continuous lines represent the BSP of all speakers of the corresponding language, the dashed lines represent the BSP of speakers of the corresponding language carrying haplogroup L2a.

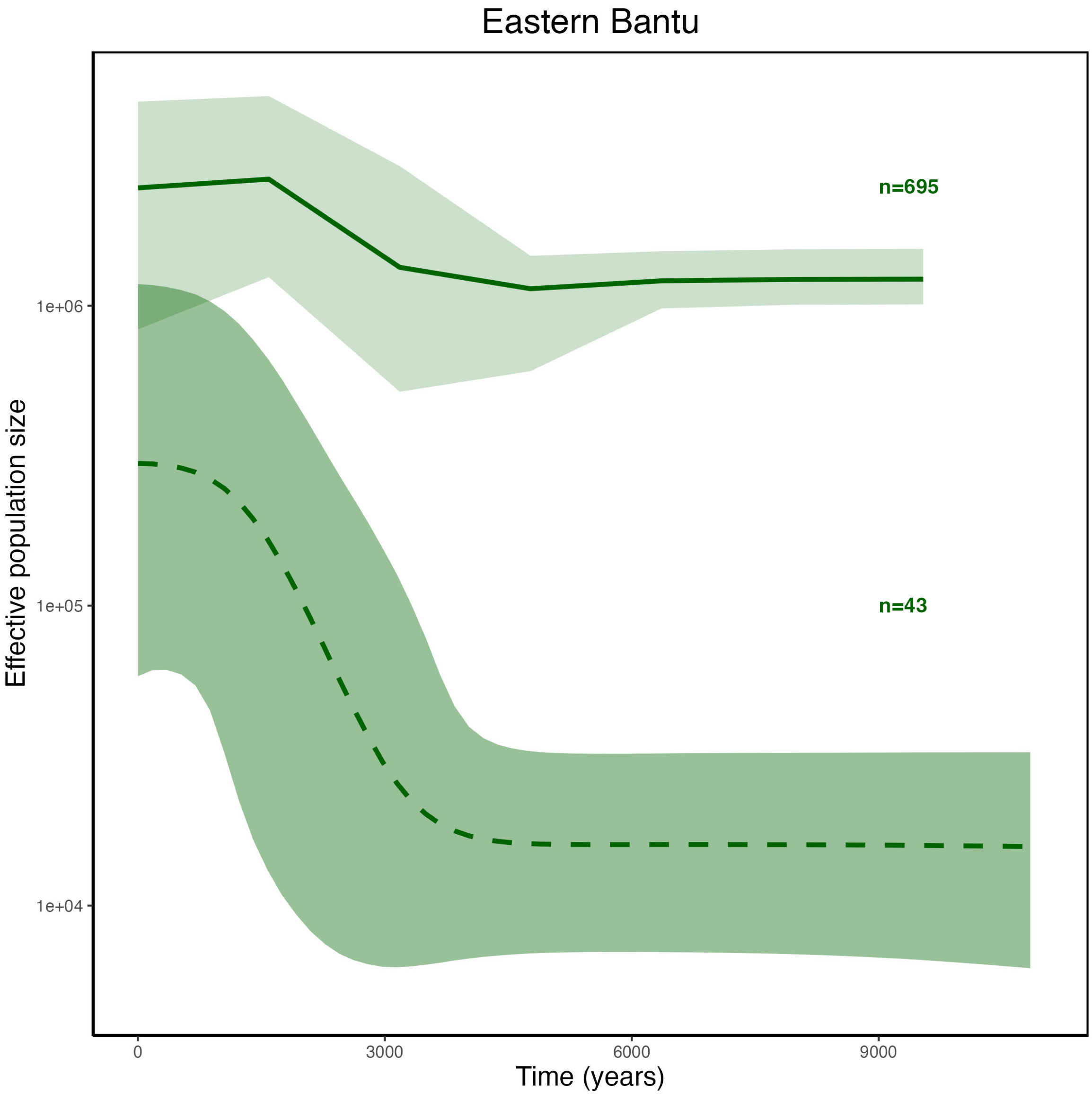


Supplementary Figure 29: Bayesian Skyline Plots showing the variation of $N_e$ through time estimated from L3b carriers of separate Bantu speaker groups. Bayesian Skyline analysis for L3b carriers in North-Western and West-Western Bantu speakers was not possible due to the low number of individuals with this haplogroup among them. The number of individuals used for each BSP is indicated next to the line in the corresponding colour. The continuous lines represent the BSP of all speakers of the corresponding language, the dashed lines represent the BSP of speakers of the corresponding language carrying haplogroup L3b.

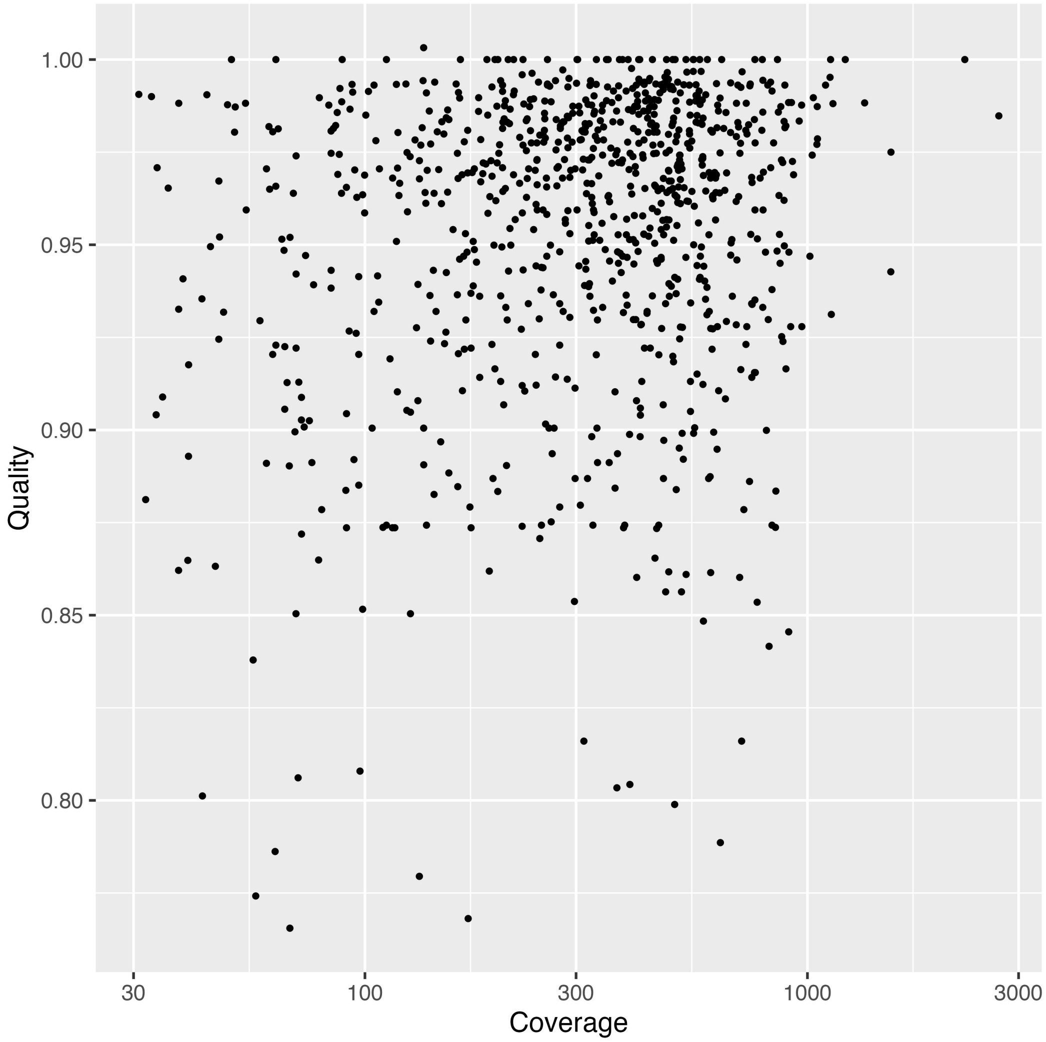


Supplementary Figure 30: HaploGrep3 quality score of all the samples in the second sequencing run that had a coverage over 30x.

## Supplementary Tables

Supplementary Table 1: Ancestry associated by literature with the mitochondrial haplogroups. References are given in the last column.

| Haplogroup | Associated ancestry | Reference |
|---|---|---|
| B4 | Asian | [103] |
| B5a | Asian | [103] |
| F1a | Asian | [103] |
| M | Asian | [103] |
| R | Asian | [103] |
| R0 | Asian | [103] |
| R5 | Asian | [103] |
| R6 | Asian | [103] |
| U2b | Asian | [103] |
| U2c | Asian | [103] |
| U2d | Asian | [104] |
| U7 | Asian | [103] |
| L0a | West-Central Africa (Bantu) | [52, 53, 105] |
| L1' | West-Central Africa (Bantu) | [106] |
| L1a | West-Central Africa (Bantu) | [54] |
| L1b | West-/Central Africa (Bantu) | [107] |
| L1c | West-Central Africa (Bantu) | [105] |
| L1e | West-Central Africa (Bantu) | [58, 59] |
| L2 | West-Central Africa (Bantu) | [58, 59] |
| L2' | West-Central Africa (Bantu) | [106] |
| L2a | West-Central Africa (Bantu) | [105] |
| L2b | West-Central Africa (Bantu) | [62] |
| L2c | West-Central Africa (Bantu) | [62] |
| L2d | West-Central Africa (Bantu) | [62] |
| L2e | West-Central Africa (Bantu) | [61] |
| L3b | West-Central Africa (Bantu) | [57] |
| L3d | West-Central Africa (Bantu) | [62] |
| L3e | West-Central Africa (Bantu) | [58, 59, 105, 54] |
| L0b | East African | [51] |
| L0f | East African | [108] |
| L3a | East African | [108] |
| L3c | East African | [67] |
| L3f | East African | [108] |
| L3h | East African | [108] |
| L3i | East African | [108] |
| L3x | East African | [108] |
| L4 | East African | [108] |
| L4 | East African | [109] |
| L4a | East African | [109] |
| L4b | East African | [108] |
| L5 | East African | [108] |
| L5a | East African | [108] |
| L5b | East African | [108] |
| L6a | East African | [109] |
| L6b | East African | [109] |
| M1a | East African | [108] |
| M1b | East African | [110] |
| R0a | East African | [111] |
| B4a | East Asian | [103] |
| B4b | East Asian | [103] |
| B5b | East Asian | [112] |
| H | European | [103] |
| H1a | European | [113] |
| H1c | European | [113] |
| H5a | European | [113] |
| H53 | European | [3] |
| HV | European | [103] |
| HV+ | European | [103] |
| HV1 | European | [103] |
| I2' | European | [3] |
| J1c | European | [114] |
| J1d | European | [3] |
| J2a | European | [3] |
| K | European | [103] |
| K1a | European | [115] |
| N | European | [3] |
| N1a | European | [3] |
| N1b | European | [3] |
| T2b | European | [103] |
| U2e | European | [103] |
| U3a | European | [3] |
| U4' | European | [3] |
| U5a1 | European | [103] |
| U8b | European | [116] |
| V | European | [3] |
| L0d | KhoeSan | [1, 50, 8] |
| L0k | KhoeSan | [1, 50, 8] |
| H1v | North African | [117] |
| L3k | North African | [106] |
| U6a | North African | [118] |
| U6b | North African | [118] |
| U6c | North African | [118] |
| U6d | North African | [118] |
| L0 | Other | [1] |
| L3 | Other | [106] |
| L3' | Other | [106] |
| E1a | South Asian | [119] |
| M18 | South Asian | [120] |
| M2a | South Asian | [120] |
| M2b | South Asian | [120] |
| M33 | South Asian | [120] |
| M42 | South Asian | [121] |
| M5a | South Asian | [120] |
| M6a | South Asian | [3] |
| U2a | South Asian | [122] |
| U7a | South Asian | [122] |
| L0g | Unknown | No reference |

Supplementary Table 2: Time to the most recent common ancestor (TMRCA) of all individuals belonging to certain groups. Reported 95% confidence intervals reflect the phylogenetic uncertainties. The second column shows TMRCAs estimated with a mutation rate of $2.285 \times 10^{-8}$ [60], as reported by BEAST. The third column shows recalibrated estimates following the correction proposed by Soares *et al.* (2009) [75] on the original BEAST TMRCA (column 2).

| Group | TMRCA (95% CI) (kya) ($2.285 \times 10^{-8}$) | Recalibrated TMRCA (95% CI) (kya) (Soares *et al.*, 2009)[75] |
|---|---|---|
| All modern humans | 132.4 (117.4 - 147.8) | 165.6 (144.6 - 187.3) |
| L0d | 95.5 (76.0 - 117.7) | 114.6 (88.6 - 145.1) |
| L0f | 81.7 (70.4 - 93.3) | 96.4 (82.5 - 110.4) |
| L0k | 64.7 (47.5 - 82.8) | 74.0 (52.5 - 97.6) |
| L0a | 73.2 (61.1 - 86.0) | 86.0 (69.0 - 103.4) |
| L1c | 83.9 (73.5 - 95.2) | 99.9 (85.3 - 114.2) |
| L2a | 58.4 (48.9 - 68.4) | 66.0 (54.2 - 78.8) |
| L3e | 36.1 (30.0 - 42.4) | 39.0 (32.0 - 46.5) |

Supplementary Table 3: Ethical clearance and sample permission details for newly generated study samples. Ethical clearance and sample permission information for the newly generated samples in this study, including associated countries, Swedish reference numbers, local reference numbers, and the corresponding local institutions.

| **Collection name** | **Country** | **Swedish reference number** | **Local reference number** | **Local institution** |
|---|---|---|---|---|
| DRC samples | DRC | Dnr 2019-05244 and Dnr 2021-01448 | Nr 091/CAB/MIN/-CA/PKB/2018 | Minister of Arts and Culture of the DRC |
| Ethiopian samples | Ethiopia | Dnr 2021-01448 | 310/169/2018 | The Federal Democratic Republic of Ethiopia Ministry of Science and Technology |
| Khoe-San and Bantu samples from Africa | Zimbabwe, South Africa, Namibia, Uganda, Zambia, Zanzibar | Dnr 2021-01448 | M180654, M180655, M180656 | Human Reearch Ethics Committee from Witwatersrand University |
| Khoe-San and Bantu samples from Botswana | Botswana | Dnr 2019-00115 | Permission obtained from the Botswana government in accordance with national regulations at the time of sampling | The Botswana Governmental Office |
| Sahel samples | Chad, Sudan | Dnr 2021-01448 | 2016-07 | Institutional research board of Charles University, Prague |
| South African samples | South Africa | Dnr 2021-01448 | EC160429-024 (Health Ethics reference 259/2016) | Faculty of Natural and Agricultural Sciences University of Pretoria |
| Cameroon samples | Cameroon | Not applicable | CBI/397/ERCC/ CAMBIN | Ethics Review and Consultancy Committee (ERCC), Cameroon Bioethics Initiative (CAMBIN) |
| Coloured, Khoe-San and Khoe-San descendants | South Africa | Dnr 2021–01448 | M980553 (renewals M050902, M090576, M1604104) | Human Reearch Ethics Committee from Witwatersrand University |
| Togo samples | Togo | Dnr 2021-04382 | N097/MESR/SG/-DRST/19 | Ministry of higher education and research |

Supplementary Table 4: Primer sequences used for full mtDNA amplification using long-range PCR. The length of the primers, including the barcode is given in the last column.

| Primer | Sequence | Length including barcode |
|---|---|---|
| FW | ATCAACCATTAACCTTCCCTCTACACTTATCATCTTCAC | 55 bp |
| RV | ATTGCTAGGGTGGCGCTTCCAATTAGGTGC | 46 bp |

Supplementary Table 5: Barcoded primers used for amplification of the mtDNA sequences. The number of forward (32) and reverse (32) primers could create 1,024 unique combinations.

| Primer | Sequence |
|---|---|
| FULL_MT_1_FW | CACATATCAGAGTGCGATCAACCATTAACCTTCCCTCTACACTTATCATCTTCAC |
| FULL_MT_2_FW | ACACACAGACTGTGAGATCAACCATTAACCTTCCCTCTACACTTATCATCTTCAC |
| FULL_MT_3_FW | ACACATCTCGTGAGAGATCAACCATTAACCTTCCCTCTACACTTATCATCTTCAC |
| FULL_MT_4_FW | CACGCACACACGCGCGATCAACCATTAACCTTCCCTCTACACTTATCATCTTCAC |
| FULL_MT_5_FW | CACTCGACTCTCGCGTATCAACCATTAACCTTCCCTCTACACTTATCATCTTCAC |
| FULL_MT_6_FW | CATATATATCAGCTGTATCAACCATTAACCTTCCCTCTACACTTATCATCTTCAC |
| FULL_MT_7_FW | TCTGTATCTCTATGTGATCAACCATTAACCTTCCCTCTACACTTATCATCTTCAC |
| FULL_MT_8_FW | ACAGTCGAGCGCTGCGATCAACCATTAACCTTCCCTCTACACTTATCATCTTCAC |
| FULL_MT_9_FW | ACACACGCGAGACAGAATCAACCATTAACCTTCCCTCTACACTTATCATCTTCAC |
| FULL_MT_10_FW | ACGCGCTATCTCAGAGATCAACCATTAACCTTCCCTCTACACTTATCATCTTCAC |
| FULL_MT_11_FW | CTATACGTATATCTATATCAACCATTAACCTTCCCTCTACACTTATCATCTTCAC |
| FULL_MT_12_FW | ACACTAGATCGCGTGTATCAACCATTAACCTTCCCTCTACACTTATCATCTTCAC |
| FULL_MT_13_FW | CTCTCGCATACGCGAGATCAACCATTAACCTTCCCTCTACACTTATCATCTTCAC |
| FULL_MT_14_FW | CTCACTACGCGCGCGTATCAACCATTAACCTTCCCTCTACACTTATCATCTTCAC |
| FULL_MT_15_FW | CGCATGACACGTGTGTATCAACCATTAACCTTCCCTCTACACTTATCATCTTCAC |
| FULL_MT_16_FW | CATAGAGAGATAGTATATCAACCATTAACCTTCCCTCTACACTTATCATCTTCAC |
| FULL_MT_17_FW | CACACGCGCGCTATATATCAACCATTAACCTTCCCTCTACACTTATCATCTTCAC |
| FULL_MT_18_FW | TCACGTGCTCACTGTGATCAACCATTAACCTTCCCTCTACACTTATCATCTTCAC |
| FULL_MT_19_FW | ACACACTCTATCAGATATCAACCATTAACCTTCCCTCTACACTTATCATCTTCAC |
| FULL_MT_20_FW | CACGACACGACGATGTATCAACCATTAACCTTCCCTCTACACTTATCATCTTCAC |
| FULL_MT_21_FW | CTATACATAGTGATGTATCAACCATTAACCTTCCCTCTACACTTATCATCTTCAC |
| FULL_MT_22_FW | CACTCACGTGTGATATATCAACCATTAACCTTCCCTCTACACTTATCATCTTCAC |
| FULL_MT_23_FW | CAGAGAGATATCTCTGATCAACCATTAACCTTCCCTCTACACTTATCATCTTCAC |
| FULL_MT_24_FW | CATGTAGAGCAGAGAGATCAACCATTAACCTTCCCTCTACACTTATCATCTTCAC |
| FULL_MT_25_FW | CGCGACACGCTCGCGCATCAACCATTAACCTTCCCTCTACACTTATCATCTTCAC |
| FULL_MT_26_FW | CACAGAGACACGCACAATCAACCATTAACCTTCCCTCTACACTTATCATCTTCAC |
| FULL_MT_27_FW | CTCACACTCTCTCACAATCAACCATTAACCTTCCCTCTACACTTATCATCTTCAC |
| FULL_MT_28_FW | CTCTGCTCTGACTCTCATCAACCATTAACCTTCCCTCTACACTTATCATCTTCAC |
| FULL_MT_29_FW | TATATATGTCTATAGAATCAACCATTAACCTTCCCTCTACACTTATCATCTTCAC |
| FULL_MT_30_FW | TCTCTCTATCGCGCTCATCAACCATTAACCTTCCCTCTACACTTATCATCTTCAC |
| FULL_MT_31_FW | GATGTCTGAGTGTGTGATCAACCATTAACCTTCCCTCTACACTTATCATCTTCAC |
| FULL_MT_32_FW | GAGACTAGAGATAGTGATCAACCATTAACCTTCCCTCTACACTTATCATCTTCAC |
| FULL_MT_65_RV | GCGCTCTCTCACATACATTGCTAGGGTGGCGCTTCCAATTAGGTGC |
| FULL_MT_66_RV | TATATGCTCTGTGTGAATTGCTAGGGTGGCGCTTCCAATTAGGTGC |
| FULL_MT_67_RV | CTCTATATATCTCGTCATTGCTAGGGTGGCGCTTCCAATTAGGTGC |
| FULL_MT_68_RV | AGAGAGCTCTCTCATCATTGCTAGGGTGGCGCTTCCAATTAGGTGC |
| FULL_MT_69_RV | GCGAGAGTGAGACGCAATTGCTAGGGTGGCGCTTCCAATTAGGTGC |
| FULL_MT_70_RV | TGCTCTCGTGTACTGTATTGCTAGGGTGGCGCTTCCAATTAGGTGC |
| FULL_MT_71_RV | AGCGCTGCGACACGCGATTGCTAGGGTGGCGCTTCCAATTAGGTGC |
| FULL_MT_72_RV | AGACGCGAGCGCGTAGATTGCTAGGGTGGCGCTTCCAATTAGGTGC |
| FULL_MT_73_RV | GCGTGTGTCGAGTGTAATTGCTAGGGTGGCGCTTCCAATTAGGTGC |
| FULL_MT_74_RV | TGTACGCTCTCTATATATTGCTAGGGTGGCGCTTCCAATTAGGTGC |
| FULL_MT_75_RV | TAGAGAGCGTCGCGTGATTGCTAGGGTGGCGCTTCCAATTAGGTGC |
| FULL_MT_76_RV | GTGCACTCGCGCTCTCATTGCTAGGGTGGCGCTTCCAATTAGGTGC |
| FULL_MT_77_RV | TATCTCTCGAGTCGCGATTGCTAGGGTGGCGCTTCCAATTAGGTGC |
| FULL_MT_78_RV | CTCACACATACACGTCATTGCTAGGGTGGCGCTTCCAATTAGGTGC |
| FULL_MT_79_RV | ATAGTACACTCTGTGTATTGCTAGGGTGGCGCTTCCAATTAGGTGC |
| FULL_MT_80_RV | TATCTCTGTAGAGTCTATTGCTAGGGTGGCGCTTCCAATTAGGTGC |
| FULL_MT_81_RV | GATATATATGTGTGTAATTGCTAGGGTGGCGCTTCCAATTAGGTGC |
| FULL_MT_82_RV | GTGACACACAGAGCACATTGCTAGGGTGGCGCTTCCAATTAGGTGC |
| FULL_MT_83_RV | ATATGACATACACGCAATTGCTAGGGTGGCGCTTCCAATTAGGTGC |
| FULL_MT_84_RV | CGTCTCTCGTCTGTGCATTGCTAGGGTGGCGCTTCCAATTAGGTGC |
| FULL_MT_85_RV | ACACAGTAGAGCGAGCATTGCTAGGGTGGCGCTTCCAATTAGGTGC |
| FULL_MT_86_RV | GTCGCGCATAGAGCGCATTGCTAGGGTGGCGCTTCCAATTAGGTGC |
| FULL_MT_87_RV | CTATCTAGCACTCACAATTGCTAGGGTGGCGCTTCCAATTAGGTGC |
| FULL_MT_88_RV | CGTGTCACTCTGCGTGATTGCTAGGGTGGCGCTTCCAATTAGGTGC |
| FULL_MT_89_RV | CGCGCGAGTATCTCGTATTGCTAGGGTGGCGCTTCCAATTAGGTGC |
| FULL_MT_90_RV | AGCACACATATAGCGCATTGCTAGGGTGGCGCTTCCAATTAGGTGC |
| FULL_MT_91_RV | GTATATATATACGTCTATTGCTAGGGTGGCGCTTCCAATTAGGTGC |
| FULL_MT_92_RV | TCTCACGAGAGCGCACATTGCTAGGGTGGCGCTTCCAATTAGGTGC |
| FULL_MT_93_RV | TAGATGCGAGAGTAGAATTGCTAGGGTGGCGCTTCCAATTAGGTGC |
| FULL_MT_94_RV | ATAGCGACATCTCTCTATTGCTAGGGTGGCGCTTCCAATTAGGTGC |
| FULL_MT_95_RV | GCACGATGTCAGCGCGATTGCTAGGGTGGCGCTTCCAATTAGGTGC |
| FULL_MT_96_RV | TGTGCTCTCTACACAGATTGCTAGGGTGGCGCTTCCAATTAGGTGC |

Supplementary Table 6: Settings for BEAST analyses by group: number of individuals, MCMC iterations, replicates, burn-in, and total iterations used for for Bayesian phylogenies. The table presents the configurations used for BEAST (Bayesian Evolutionary Analysis Sampling Trees) analyses, organized by language groups. Each group is characterized by the number of individuals within it. The BEAST analyses entail multiple runs, each involving a specific number of Markov Chain Monte Carlo (MCMC) iterations, conducted in replicate. These replicates are subsequently merged to enhance the accuracy of Bayesian phylogeny inferences. The table also notes the 'burn-in' period discarded during the analysis and the total number of iterations employed in generating the Bayesian Skyline Plot.

| Group (language/haplogroup) | Number of individuals | Iterations (million) | Replicate runs performed | Discarded burn-in (million) | Total iterations used for BSP (million) |
|---|---|---|---|---|---|
| Khoisan, only L0d and L0k | 609 | 20 | 50 | 2 | 900 |
| Khoisan | 777 | 20 | 50 | 2 | 900 |
| Niger-Congo (non-Bantu) and Mande | 830 | 20 | 60 | 2 | 1080 |
| Niger-Congo (with Bantu) and Mande (subset) | 500 | 20 | 50 | 2 | 900 |
| Bantu (subset) | 500 | 20 | 50 | 2 | 900 |
| Afro-Asiatic | 520 | 20 | 50 | 2 | 900 |
| Nilo-Saharan | 121 | 100 | 20 | 10 | 1800 |
| Afrikaans, only L0d and L0k | 144 | 100 | 20 | 10 | 1800 |
| Afrikaans, only Indo-European haplogroups | 21 | 100 | 10 | 10 | 900 |
| Afrikaans | 197 | 100 | 20 | 10 | 1800 |
| North-Western Bantu | 94 | 100 | 10 | 10 | 900 |
| West-Western Bantu | 313 | 100 | 20 | 10 | 1800 |
| South Western Bantu | 784 | 20 | 50 | 2 | 900 |
| Eastern Bantu | 695 | 20 | 50 | 2 | 900 |
| North-Western Bantu L3e | 20 | 100 | 10 | 10 | 900 |
| West-Western Bantu L3e | 71 | 100 | 10 | 10 | 900 |
| South Western Bantu L3e | 154 | 100 | 20 | 10 | 1800 |
| Eastern Bantu L3e | 89 | 100 | 10 | 10 | 900 |
| North-Western Bantu L1c | 26 | 100 | 10 | 10 | 900 |
| West-Western Bantu L1c | 56 | 100 | 10 | 10 | 900 |
| South Western Bantu L1c | 132 | 100 | 20 | 10 | 1800 |
| Eastern Bantu L1c | 83 | 100 | 10 | 10 | 900 |
| North-Western Bantu L0a | 7 | 100 | 10 | 10 | 900 |
| West-Western Bantu L0a | 39 | 100 | 10 | 10 | 900 |
| South Western Bantu L0a | 103 | 100 | 10 | 10 | 900 |
| Eastern Bantu L0a | 139 | 100 | 20 | 10 | 1800 |
| North-Western Bantu L2a | 15 | 100 | 10 | 10 | 900 |
| West-Western Bantu L2a | 47 | 100 | 10 | 10 | 900 |
| South Western Bantu L2a | 86 | 100 | 10 | 10 | 900 |
| Eastern Bantu L2a | 89 | 100 | 10 | 10 | 900 |
| North-Western Bantu L3b | 5 | 100 | 10 | 10 | 900 |
| West-Western Bantu L3b | 10 | 100 | 10 | 10 | 900 |
| South Western Bantu L3b | 14 | 100 | 10 | 10 | 900 |
| Eastern Bantu L3b | 43 | 100 | 10 | 10 | 900 |

# References


[1] Behar, D. M., Villems, R., Soodyall, H., Blue-Smith, J., Pereira, L., Metspalu, E., Scozzari, R., Makkan, H., Tzur, S., Comas, D., Bertranpetit, J., Quintana-Murci, L., Tyler-Smith, C., Wells, R. S., and Rosset, S. (2008). The Dawn of Human Matrilineal Diversity. *The American Journal of Human Genetics* **82**(5), 1130–1140. doi:10.1016/j.ajhg.2008.04.002.

[2] Rosenberg, N. A., Pritchard, J. K., Weber, J. L., Cann, H. M., Kidd, K. K., Zhivotovsky, L. A., and Feldman, M. W. (2002). Genetic structure of human populations. *Science* **298**(5602), 2381–2385. doi:10.1126/science.1078311.

[3] Underhill, P. A. and Kivisild, T. (2007). Use of Y Chromosome and Mitochondrial DNA Population Structure in Tracing Human Migrations. *Annual Review of Genetics* **41**, 539–564. doi:10.1146/annurev.genet.41.110306.130407.

[4] Vicente, M., Jakobsson, M., Ebbesen, P., and Schlebusch, C. M. (2019). Genetic Affinities among Southern Africa Hunter-Gatherers and the Impact of Admixing Farmer and Herder Populations. *Molecular Biology and Evolution* **36**(9), 1849–1861. doi:10.1093/molbev/msz089.

[5] Fortes-Lima, C. A., Burgarella, C., Hammarén, R., Eriksson, A., Vicente, M., Jolly, C., Semo, A., Gunnink, H., Pacchiarotti, S., Mundeke, L., Matonda, I., Muluwa, J. K., Coutros, P., Nyambe, T. S., Cikomola, J. C., Coetzee, V., de Castro, M., Ebbesen, P., Delanghe, J., Stoneking, M., Barham, L., Lombard, M., Meyer, A., Steyn, M., Malmström, H., Rocha, J., Soodyall, H., Pakendorf, B., Bostoen, K., and Schlebusch, C. M. (2024). The genetic legacy of the expansion of Bantu-speaking peoples in Africa. *Nature* **625**(7995), 540–547. doi:10.1038/s41586-023-06770-6.

[6] Pakendorf, B. and Stoneking, M. (2005). Mitochondrial DNA and human evolution. *Annual Review of Genomics and Human Genetics* **6**, 165–183. doi:10.1146/annurev.genom.6.080604.162249.

[7] Černý, V., Salas, A., Hájek, M., Žaloudková, M., and Brdička, R. (2007). A Bidirectional Corridor in the Sahel-Sudan Belt and the Distinctive Features of the Chad Basin Populations: A History Revealed by the Mitochondrial DNA Genome. *Annals of Human Genetics* **71**(4), 433–452. doi:10.1111/j.1469-1809.2006.00339.x.

[8] Schlebusch, C. M., Lombard, M., and Soodyall, H. (2013). MtDNA control region variation affirms diversity and deep sub-structure in populations from southern Africa. *BMC Evol Biol* **13**(1), 56. doi:10.1186/1471-2148-13-56.

[9] Schlebusch, C. M., de Jongh, M., and Soodyall, H. (2011). Different contributions of ancient mitochondrial and Y-chromosomal lineages in 'Karretjie people'of the Great Karoo in South Africa. *Journal of Human Genetics* **56**(9), 623–630. doi:10.1038/jhg.2011.71.

[10] Tishkoff, S. A., Gonder, M. K., Henn, B. M., Mortensen, H., Knight, A., Gignoux, C., Fernandopulle, N., Lema, G., Nyambo, T. B., Ramakrishnan, U., Reed, F. A.,

and Mountain, J. L. (2007). History of click-speaking populations of Africa inferred from mtDNA and Y chromosome genetic variation. *Molecular Biology and Evolution* **24**(10), 2180–2195. doi:10.1093/molbev/msm155.

[11] Pereira, L., Černý, V., Cerezo, M., Silva, N. M., Hájek, M., Vašíková, A., Kujanová, M., Brdička, R., and Salas, A. (2010). Linking the sub-Saharan and West Eurasian gene pools: maternal and paternal heritage of the Tuareg nomads from the African Sahel. *European Journal of Human Genetics* **18**(8), 915–923. doi:10.1038/ejhg.2010.21.

[12] Sanger, F., Nicklen, S., and Coulson, A. R. (1977). DNA sequencing with chain-terminating inhibitors. *Proceedings of the National Academy of Sciences* **74**(12), 5463–5467. doi:10.1073/pnas.74.12.5463.

[13] Anderson, S., Bankier, A. T., Barrell, B. G., de Bruijn, M. H. L., Coulson, A. R., Drouin, J., Eperon, I. C., Nierlich, D. P., Roe, B. A., Sanger, F., Schreier, P. H., Smith, A. J. H., Staden, R., and Young, I. G. (1981). Sequence and organization of the human mitochondrial genome. *Nature* **290**(5806), 457–465. doi:10.1038/290457a0.

[14] Vossen, R. H. A. M. and Buermans, H. P. J. (2017). *Full-Length Mitochondrial-DNA Sequencing on the PacBio RSII.* Springer New York, New York, NY.

[15] Cann, R. L., Stoneking, M., and Wilson, A. C. (1987). MtDNA and human evolution. *Nature* **325**(6099), 31–36. doi:10.1038/325031a0.

[16] Ingman, M., Kaessmann, H., Pääbo, S., and Gyllensten, U. (2000). Mitochondrial genome variation and the origin of modern humans. *Nature* **408**(6813), 708–713. doi:10.1038/35047064.

[17] Maca-Meyer, N., González, A. M., Pestano, J., Flores, C., Larruga, J., and Cabrera, V. M. (2003). Mitochondrial DNA transit between West Asia and North Africa inferred from U6 phylogeography. *BMC Genetics* **4**(1), 15. doi:10.1186/1471-2156-4-15.

[18] Pennarun, E., Kivisild, T., Metspalu, E., Metspalu, M., Reisberg, T., Moisan, J.-P., Behar, D. M., Jones, S. C., and Villems, R. (2012). Divorcing the Late Upper Palaeolithic demographic histories of mtDNA haplogroups M1 and U6 in Africa. *BMC Evol Biol* **12**(1), 234. doi:10.1186/1471-2148-12-234.

[19] González, A. M., Larruga, J., Abu-Amero, K. K., Shi, Y., Pestano, J., and Cabrera, V. M. (2007). Mitochondrial lineage M1 traces an early human backflow to Africa. *BMC Genomics* **8**, 223. doi:10.1186/1471-2164-8-223.

[20] Tishkoff, S. A., Reed, F. A., Friedlaender, F. R., Ehret, C., Ranciaro, A., Froment, A., Hirbo, J. B., Awomoyi, A. A., Bodo, J.-M., Doumbo, O., Ibrahim, M., Juma, A. T., Kotze, M. J., Lema, G., Moore, J. H., Mortensen, H., Nyambo, T. B., Omar, S. A., Powell, K., Pretorius, G. S., Smith, M. W., Thera, M. A., Wambebe, C., Weber, J. L., and Williams, S. M. (2009). The genetic structure and history of Africans and African Americans. *Science* **324**(5930), 1035–1044. doi:10.1126/science.1172257.

[21] de Filippo, C., Bostoen, K., Stoneking, M., and Pakendorf, B. (2012). Bringing together linguistic and genetic evidence to test the Bantu expansion. *Proceedings of the Royal Society B: Biological Sciences* **279**(1741), 3256–3263. doi:10.1098/rspb.2012.0318.

[22] Greenberg, J. H. (1963). The Languages of Africa. *International Journal of American Linguistics* **29**(1).

[23] Güldemann, T. (2018). *The Languages and Linguistics of Africa.* De Gruyter Mouton, Berlin, Boston.

[24] Dimmendaal, G. (2008). Language Ecology and Linguistic Diversity on the African Continent. *Language and Linguistics Compass* **2**, 840–858. doi:10.1111/j.1749-818X.2008.00085.x.

[25] Fan, S., Spence, J. P., Feng, Y., Hansen, M. E. B., Terhorst, J., Beltrame, M. H., Ranciaro, A., Hirbo, J., Beggs, W., Thomas, N., Nyambo, T., Mpoloka, S. W., Mokone, G. G., Njamnshi, A., Folkunang, C., Meskel, D. W., Belay, G., Song, Y. S., and Tishkoff, S. A. (2023). Whole-genome sequencing reveals a complex African population demographic history and signatures of local adaptation. *Cell* **186**(5), 923–939. doi:10.1016/j.cell.2023.01.042.

[26] Güldemann, T. and Fehn, A.-M., editors. (2014). *Beyond 'Khoisan': Historical relations in the Kalahari Basin.* John Benjamins.

[27] Schlebusch, C. (2010). Issues raised by use of ethnic-group names in genome study. *Nature* **464**(7288), 487; author reply 487.

[28] Gronau, I., Hubisz, M. J., Gulko, B., Danko, C. G., and Siepel, A. (2011). Bayesian inference of ancient human demography from individual genome sequences. *Nature Genetics* **43**(10), 1031–1034. doi:10.1038/ng.937.

[29] Schlebusch, C. M., Skoglund, P., Sjödin, P., Gattepaille, L. M., Hernandez, D., Jay, F., Li, S., De Jongh, M., Singleton, A., Blum, M. G., Soodyall, H., and Jakobsson, M. (2012). Genomic variation in seven Khoe-San groups reveals adaptation and complex African history. *Science* **338**(6105), 374–379. doi:10.1126/science.1227721.

[30] Schlebusch, C. M., Malmström, H., Günther, T., Sjödin, P., Coutinho, A., Edlund, H., Munters, A. R., Vicente, M., Steyn, M., Soodyall, H., Lombard, M., and Jakobsson, M. (2017). Southern African ancient genomes estimate modern human divergence to 350,000 to 260,000 years ago. *Science* **358**(6363), 652–655. doi:10.1126/science.aao6266.

[31] Veeramah, K. R., Wegmann, D., Woerner, A., Mendez, F. L., Watkins, J. C., Destro-Bisol, G., Soodyall, H., Louie, L., and Hammer, M. F. (2011). An Early Divergence of KhoeSan Ancestors from Those of Other Modern Humans Is Supported by an ABC-Based Analysis of Autosomal Resequencing Data. *Molecular Biology and Evolution* **29**(2), 617–630. doi:10.1093/molbev/msr212.

[32] Bostoen, K., Coutros, P., and Schlebusch, C. Niger-congo archaeolinguistics, including bantu. In *Oxford Handbook of Archaeology and Language,* Robbeets, M. and Hudson, M., editors, 592–618. Oxford: Oxford University Press (2025).

[33] Lewis, M. P., editor. (2009). *Ethnologue: Languages of the World.* SIL International, Dallas, TX, USA, sixteenth edition.

[34] Nurse, D. Bantu Languages. In *Encyclopedia of Language and Linguistics (Second Edition),* Brown, K., editor, 679–685. Elsevier, Oxfordsecond edition edition (2006).

[35] Li, S., Schlebusch, C., and Jakobsson, M. (2014). Genetic variation reveals large-scale population expansion and migration during the expansion of Bantu-speaking peoples. *Proceedings of the Royal Society B: Biological Sciences* **281**(1793), 20141448. doi:10.1098/rspb.2014.1448.

[36] Blench, R. (2006). *Archaeology, language, and the African past.* AltaMira Press.

[37] Barbieri, C., Whitten, M., Beyer, K., Schreiber, H., Li, M., and Pakendorf, B. (2012). Contrasting maternal and paternal histories in the linguistic context of Burkina Faso. *Mol Biol Evol* **29**(4), 1213–1223. doi:10.1093/molbev/msr291.

[38] Blench, R. (2017). *Africa over the last 12000 years: how we can interpret the interface of archaeology, linguistics and genetics.* McDonald Institute for Archaeological Research.

[39] Schlebusch, C. M. and Jakobsson, M. (2018). Tales of Human Migration, Admixture, and Selection in Africa. *Annual Review of Genomics and Human Genetics* **19**(1), 405–428. doi:10.1146/annurev-genom-083117-021759.

[40] Bostoen, K. The Bantu Expansion. In *Oxford Research Encyclopedia of African History.* Oxford University Press (2018).

[41] Bostoen, K. The Bantu Expansion: Some facts and fiction. In *Language Dispersal, Diversification, and Contact.* Oxford University Press (2020).

[42] Bostoen, K. Bantu expansion. In *The encyclopedia of ancient history: Asia and Africa,* Potts, Daniel T. and Harkness, Ethan and Neelis, Jason and McIntosh, Roderick J., editor, 1–7. Wiley (2023).

[43] de Maret, P. (2013). *Oxford Handbook of African Archaeology*, chapter Archaeologies of the Bantu Expansion, 627–643. Oxford: Oxford University Press.

[44] Heine, B. and Nurse, D. (2000). *African Languages An Introduction.* Cambridge University Press.

[45] Frajzyngier, Z. (2018). *Afroasiatic Languages.* Cambridge University Press.

[46] Salas, A., Richards, M., De la Fe, T., Lareu, M. V., Sobrino, B., Sánchez-Diz, P., Macaulay, V., and Carracedo, Á. (2002). The making of the African mtDNA landscape. *American Journal of Human Genetics* **71**(5), 1082–1111. doi:10.1086/344348.

[47] Cerný, V., Fernandes, V., Costa, M. D., Hájek, M., Mulligan, C. J., and Pereira, L. (2009). Migration of Chadic speaking pastoralists within Africa based on population structure of Chad Basin and phylogeography of mitochondrial L3f haplogroup. *BMC Evol Biol* **9**, 63. doi:10.1186/1471-2148-9-63.

[48] Podgorná, E., Soares, P., Pereira, L., and Cerný, V. (2013). The genetic impact of the lake chad basin population in North Africa as documented by mitochondrial diversity and internal variation of the L3e5 haplogroup. *Ann Hum Genet* **77**(6), 513–523. doi:10.1111/ahg.12040.

[49] Knight, A., Underhill, P. A., Mortensen, H. M., Zhivotovsky, L. A., Lin, A. A., Henn, B. M., Louis, D., Ruhlen, M., and Mountain, J. L. (2003). African Y Chromosome and mtDNA Divergence Provides Insight into the History of Click Languages. *Current Biology* **13**(6), 464–473. doi:10.1016/S0960-9822(03)00130-1.

[50] Barbieri, C., Vicente, M., Rocha, J., Mpoloka, S. W., Stoneking, M., and Pakendorf, B. (2013). Ancient substructure in early mtDNA lineages of Southern Africa. *American Journal of Human Genetics* **92**(2), 285–292. doi:10.1016/j.ajhg.2012.12.010.

[51] Rito, T., Richards, M. B., Fernandes, V., Alshamali, F., Cerny, V., Pereira, L., and Soares, P. (2013). The first modern human dispersals across Africa. *PLoS ONE* **8**(11). doi:10.1371/journal.pone.0080031.

[52] Bandelt, H. J., Forster, P., Sykes, B. C., and Richards, M. B. (1995). Mitochondrial portraits of human populations using median networks. *Genetics* **141**(2), 743–753. doi:10.1093/genetics/141.2.743.

[53] Chen, Y. S., Torroni, A., Excoffier, L., Santachiara-Benerecetti, A. S., and Wallace, D. C. (1995). Analysis of mtDNA variation in African populations reveals the most ancient of all human continent-specific haplogroups. *American Journal of Human Genetics* **57**(1), 133–149.

[54] Pereira, L., Macaulay, V., Torroni, A., Scozzari, R., Prata, M. J., and Amorim, A. (2001). Prehistoric and historic traces in the mtDNA of Mozambique: Insights into the Bantu expansions and the slave trade. *Annals of Human Genetics* **65**(Pt 5), 439–458. doi:10.1046/j.1469-1809.2001.6550439.x.

[55] Beleza, S., Gusmão, L., Amorim, A., Carracedo, A., and Salas, A. (2005). The genetic legacy of western Bantu migrations. *Human Genetics* **117**(4), 366–375. doi:10.1007/s00439-005-1290-3.

[56] Rando, J., Pinto, F., Gonźlez, A., Hernández, M., Larruga, J., Cabrera, V., and Bandelt, H. (1998). Mitochondrial DNA analysis of Northwest African populations reveals genetic exchanges with European, Near-Eastern, and sub-Saharan populations. *Annals of Human Genetics* **62**(6), 531–550. doi:10.1046/j.1469-1809.1998.6260531.x.

[57] Watson, E., Forster, P., Richards, M., and Bandelt, H. J. (1997). Mitochondrial footprints of human expansions in Africa. *American Journal of Human Genetics* **61**(3), 691–704. doi:10.1086/515503.

[58] Alves-Silva, J., da Silva Santos, M., Guimarães, P. E., Ferreira, A. C., Bandelt, H. J., Pena, S. D., and Prado, V. F. (2000). The ancestry of Brazilian mtDNA lineages. *American Journal of Human Genetics* **67**(2), 444–461. doi:10.1086/303004.

[59] Bandelt, H. J., Alves-Silva, J., Guimarães, P. E., Santos, M. S., Brehm, A., Pereira, L., Coppa, A., Larruga, J. M., Rengo, C., Scozzari, R., Torroni, A., Prata, M. J., Amorim, A., Prado, V. F., and Pena, S. D. (2001). Phylogeography of the human mitochondrial haplogroup L3e: A snapshot of African prehistory and Atlantic slave trade. *Annals of Human Genetics* **65**, 549–563. doi:10.1017/S0003480001008892.

[60] Maier, P. A., Runfeldt, G., Estes, R. J., and Vilar, M. G. (2022). African mitochondrial haplogroup L7: a 100,000-year-old maternal human lineage discovered through reassessment and new sequencing. *Scientific Reports* **12**(1), 10747. doi:10.1038/s41598-022-13856-0.

[61] Silva, M., Alshamali, F., Silva, P., Carrilho, C., Mandlate, F., Jesus Trovoada, M., Černý, V., Pereira, L., and Soares, P. (2015). 60,000 years of interactions between Central and Eastern Africa documented by major African mitochondrial haplogroup L2. *Scientific Reports* **5**, 12526. doi:10.1038/srep12526.

[62] Rosa, A. and Brehm, A. (2011). African human mtDNA phylogeography at-a-glance. *Journal of Anthropological Sciences* **89**, 25–58. doi:10.4436/jass.89006.

[63] Batini, C., Coia, V., Battaggia, C., Rocha, J., Pilkington, M. M., Spedini, G., Comas, D., Destro-Bisol, G., and Calafell, F. (2007). Phylogeography of the human mitochondrial L1c haplogroup: Genetic signatures of the prehistory of Central Africa. *Molecular Phylogenetics and Evolution* **43**(2), 635–644. doi:10.1016/j.ympev.2006.09.014.

[64] Quintana-Murci, L., Quach, H., Harmant, C., Luca, F., Massonnet, B., Patin, E., Sica, L., Mouguiama-Daouda, P., Comas, D., Tzur, S., Balanovsky, O., Kidd, K. K., Kidd, J. R., van der Veen, L., Hombert, J.-M., Gessain, A., Verdu, P., Froment, A., Bahuchet, S., Heyer, E., Dausset, J., Salas, A., and Behar, D. M. (2008). Maternal traces of deep common ancestry and asymmetric gene flow between Pygmy hunter-gatherers and Bantu-speaking farmers. *Proceedings of the National Academy of Sciences* **105**(5), 1596–1601. doi:10.1073/pnas.0711467105.

[65] Gonder, M. K., Mortensen, H. M., Reed, F. A., de Sousa, A., and Tishkoff, S. A. (2006). Whole-mtDNA Genome Sequence Analysis of Ancient African Lineages. *Molecular Biology and Evolution* **24**(3), 757–768. doi:10.1093/molbev/msl209.

[66] Cerezo, M., Černý, V., Carracedo, Á., and Salas, A. (2011). New insights into the Lake Chad Basin population structure revealed by high-throughput genotyping of mitochondrial DNA coding SNPs. *PLoS ONE* **6**(4), e18682. doi:10.1371/journal.pone.0018682.

[67] Soares, P., Alshamali, F., Pereira, J. B., Fernandes, V., Silva, N. M., Afonso, C., Costa, M. D., Musilová, E., MacAulay, V., Richards, M. B., Černý, V., and Pereira, L. (2012). The expansion of mtDNA haplogroup L3

within and out of Africa. *Molecular Biology and Evolution* **29**(3), 915–927. doi:10.1093/molbev/msr245.

[68] Batai, K., Babrowski, K. B., Arroyo, J. P., Kusimba, C. M., and Williams, S. R. (2013). Mitochondrial DNA diversity in two ethnic groups in southeastern Kenya: perspectives from the northeastern periphery of the Bantu expansion. *Am J Phys Anthropol* **150**(3), 482–491. doi:10.1002/ajpa.22227.

[69] Barbieri, C., Güldemann, T., Naumann, C., Gerlach, L., Berthold, F., Nakagawa, H., Mpoloka, S. W., Stoneking, M., and Pakendorf, B. (2014). Unraveling the complex maternal history of Southern African Khoisan populations. *Am J Phys Anthropol* **153**(3), 435–448. doi:10.1002/ajpa.22441.

[70] Batini, C., Lopes, J., Behar, D. M., Calafell, F., Jorde, L. B., van der Veen, L., Quintana-Murci, L., Spedini, G., Destro-Bisol, G., and Comas, D. (2010). Insights into the Demographic History of African Pygmies from Complete Mitochondrial Genomes. *Mol Biol Evol* **28**(2), 1099–1110. doi:10.1093/molbev/msq294.

[71] Oliveira, S., Fehn, A. M., Aço, T., Lages, F., Gayà-Vidal, M., Pakendorf, B., Stoneking, M., and Rocha, J. (2018). Matriclans shape populations: Insights from the Angolan Namib Desert into the maternal genetic history of southern Africa. *Am J Phys Anthropol* **165**(3), 518–535. doi:10.1002/ajpa.23378.

[72] Behar, D. M., van Oven, M., Rosset, S., Metspalu, M., Loogväli, E.-L., Silva, N. M., Kivisild, T., Torroni, A., and Villems, R. (2012). A "Copernican" reassessment of the human mitochondrial DNA tree from its root. *Am J Hum Genet* **90**(4), 675–684.

[73] Vicente, M., Lankheet, I., Russell, T., Hollfelder, N., Coetzee, V., Soodyall, H., Jongh, M. D., and Schlebusch, C. M. (2021). Male-biased migration from East Africa introduced pastoralism into southern Africa. *BMC Biology* **19**(1), 259–16. doi:10.1186/s12915-021-01193-z.

[74] Schönherr, S., Weissensteiner, H., Kronenberg, F., and Forer, L. (2023). Haplogrep 3 - an interactive haplogroup classification and analysis platform. *Nucleic Acids Research* **51**(W1), W263–W268. doi:10.1093/nar/gkad284.

[75] Soares, P., Ermini, L., Thomson, N., Mormina, M., Rito, T., Röhl, A., Salas, A., Oppenheimer, S., Macaulay, V., and Richards, M. B. (2009). Correcting for purifying selection: an improved human mitochondrial molecular clock. *Am J Hum Genet* **84**(6), 740–759. doi:org/10.1016/j.ajhg.2009.05.001.

[76] Chan, E. K. F., Timmermann, A., Baldi, B. F., Moore, A. E., Lyons, R. J., Lee, S.-S., Kalsbeek, A. M. F., Petersen, D. C., Rautenbach, H., Förtsch, H. E. A., Bornman, M. S. R., and Hayes, V. M. (2019). Human origins in a southern African palaeo-wetland and first migrations. *Nature* **575**(7781), 185–189. doi:10.1038/s41586-019-1714-1.

[77] Lankheet, I., Hammarén, R., Alva Caballero, L. X., Larena, M., Malmström, H., Jolly, C., Soodyall, H., de Jongh, M., and Schlebusch, C. (2025). Wide-scale

geographical analysis of genetic ancestry in the south african coloured population. *BMC Biology* **23**(1), 219.

[78] Skoglund, P., Thompson, J. C., Prendergast, M. E., Mittnik, A., Sirak, K., Hajdinjak, M., Salie, T., Rohland, N., Mallick, S., Peltzer, A., Heinze, A., Olalde, I., Ferry, M., Harney, E., Michel, M., Stewardson, K., Cerezo-Román, J. I., Chiumia, C., Crowther, A., Gomani-Chindebvu, E., Gidna, A. O., Grillo, K. M., Helenius, I. T., Hellenthal, G., Helm, R., Horton, M., López, S., Mabulla, A. Z. P., Parkington, J., Shipton, C., Thomas, M. G., Tibesasa, R., Welling, M., Hayes, V. M., Kennett, D. J., Ramesar, R., Meyer, M., Pääbo, S., Patterson, N., Morris, A. G., Boivin, N., Pinhasi, R., Krause, J., and Reich, D. (2017). Reconstructing Prehistoric African Population Structure. *Cell* **171**(1), 59–71.

[79] Kivisild, T., Reidla, M., Metspalu, E., Rosa, A., Brehm, A., Pennarun, E., Parik, J., Geberhiwot, T., Usanga, E., and Villems, R. (2004). Ethiopian Mitochondrial DNA Heritage: Tracking Gene Flow Across and Around the Gate of Tears. *The American Journal of Human Genetics* **75**(5), 752–770.

[80] Kim, H. L., Ratan, A., Perry, G. H., Montenegro, A., Miller, W., and Schuster, S. C. (2014). Khoisan hunter-gatherers have been the largest population throughout most of modern-human demographic history. *Nature communications* **5**(1), 1–8. doi:10.1038/ncomms6692.

[81] Koile, E., Greenhill, S. J., Blasi, D. E., Bouckaert, R., and Gray, R. D. (2022). Phylogeographic analysis of the Bantu language expansion supports a rainforest route. *Proceedings of the National Academy of Sciences* **119**(32), e2112853119. doi:10.1073/pnas.2112853119.

[82] Vansina, J. (1995). New Linguistic Evidence and 'The Bantu Expansion'. *The Journal of African History* **36**(2), 173–195. doi:10.1017/S0021853700034101.

[83] Sengupta, D., Choudhury, A., Fortes-Lima, C., Aron, S., Whitelaw, G., Bostoen, K., Gunnink, H., Chousou-Polydouri, N., Delius, P., Tollman, S., Gómez-Olivé, F. X., Norris, S., Mashinya, F., Alberts, M., Hazelhurst, S., Schlebusch, C. M., Ramsay, M., Study, A.-G., and Consortium, H. (2021). Genetic substructure and complex demographic history of South African Bantu speakers. *Nature Communications* **12**(1), 2080. doi:10.1038/s41467-021-22207-y.

[84] Seidensticker, D., Hubau, W., Verschuren, D., Fortes-Lima, C., de Maret, P., Schlebusch, C. M., and Bostoen, K. (2021). Population collapse in Congo rainforest from 400 CE urges reassessment of the Bantu Expansion. *Science Advances* **7**(7), eabd8352. doi:10.1126/sciadv.abd8352.

[85] Choudhury, A., Aron, S., Botigué, L. R., Sengupta, D., Botha, G., Bensellak, T., Wells, G., Kumuthini, J., Shriner, D., Fakim, Y. J., Ghoorah, A. W., Dareng, E., Odia, T., Falola, O., Adebiyi, E., Hazelhurst, S., Mazandu, G., Nyangiri, O. A., Mbiyavanga, M., Benkahla, A., Kassim, S. K., Mulder, N., Adebamowo, S. N., Chimusa, E. R., Muzny, D., Metcalf, G., Gibbs, R. A., Matovu, E., Bucheton, B., Hertz-Fowler, C., Koffi, M., Macleod, A., Mumba-Ngoyi, D., Noyes, H., Nyangiri,

O. A., Simo, G., Simuunza, M., Rotimi, C., Ramsay, M., Botigué, L., Fakim, Y. J., Ghoorah, A. W., Nyangiri, O. A., Kassim, S. K., Adebamowo, S. N., Chimusa, E. R., Adeyemo, A. A., Lombard, Z., Hanchard, N. A., Adebamowo, C., Agongo, G., Boua, R. P., Oduro, A., Sorgho, H., Landouré, G., Cissé, L., Diarra, S., Samassékou, O., Anabwani, G., Matshaba, M., Joloba, M., Kekitiinwa, A., Mardon, G., Mpoloka, S. W., Kyobe, S., Mlotshwa, B., Mwesigwa, S., Retshabile, G., Williams, L., Wonkam, A., Moussa, A., Adu, D., Ojo, A., Burke, D., Salako, B. O., Nyangiri, O. A., Awadalla, P., Bruat, V., Gbeha, E., Adeyemo, A. A., Hanchard, N. A., Group, T. R., and Consortium, H. (2020). High-depth African genomes inform human migration and health. *Nature* **586**(7831), 741–748. doi:10.1038/s41586-020-2859-7.

[86] Browning, S. R. and Browning, B. L. (2015). Accurate non-parametric estimation of recent effective population size from segments of identity by descent. *Am J Hum Genet* **97**(3), 404–418. doi:org/10.1016/j.ajhg.2015.07.012å.

[87] Fu, Q., Mittnik, A., Johnson, P. L., Bos, K., Lari, M., Bollongino, R., Sun, C., Giemsch, L., Schmitz, R., Burger, J., Ronchitelli, A. M., Martini, F., Cremonesi, R. G., Svoboda, J., Bauer, P., Caramelli, D., Castellano, S., Reich, D., Pääbo, S., and Krause, J. (2013). A revised timescale for human evolution based on ancient mitochondrial genomes. *Current Biology* . doi:10.1016/j.cub.2013.02.044.

[88] Huffman, T. (2007). *Handbook to the Iron Age: The Archaeology of Pre-colonial Farming Societies in Southern Africa*. University of KwaZulu-Natal Press.

[89] Jakobsson, M., Bernhardsson, C., McKenna, J., Hollfelder, N., Vicente, M., Edlund, H., Coutinho, A., Sjödin, P., Brink, J., Zipfel, B., Malmström, H., Lombard, M., and Schlebusch, C. M. (2025). Homo sapiens-specific evolution unveiled by ancient southern african genomes. *Nature* . doi:10.1038/s41586-025-09811-4.

[90] Heller, R., Chikhi, L., and Siegismund, H. R. (2013). The confounding effect of population structure on Bayesian skyline plot inferences of demographic history. *PLoS ONE* **8**(5), e62992. doi:10.1371/journal.pone.0062992.

[91] Poplin, R., Chang, P.-C., Alexander, D., Schwartz, S., Colthurst, T., Ku, A., Newburger, D., Dijamco, J., Nguyen, N., Afshar, P. T., Gross, S. S., Dorfman, L., McLean, C. Y., and DePristo, M. A. (2018). A universal SNP and small-indel variant caller using deep neural networks. *Nat Biotechnol* **36**(10), 983–987. doi:10.1038/nbt.4235.

[92] Van der Auwera, G. A. and O'Connor, B. D. (2020). *Genomics in the cloud: using Docker, GATK, and WDL in Terra*. O'Reilly Media.

[93] Matheron, G. (1963). Principles of geostatistics. *Economic Geology* **58**(8), 1246–1266. doi:10.2113/gsecongeo.58.8.1246.

[94] Suchard, M. A., Lemey, P., Baele, G., Ayres, D. L., Drummond, A. J., and Rambaut, A. (2018). Bayesian phylogenetic and phylodynamic data integration using BEAST 1.10. *Virus Evol* **4**(1), vey016. doi:10.1093/ve/vey016.

[95] Poznik, G. D., Henn, B. M., Yee, M. C., Sliwerska, E., Euskirchen, G. M., Lin, A. A., Snyder, M., Quintana-Murci, L., Kidd, J. M., Underhill, P. A., and Bustamante, C. D. (2013). Sequencing Y chromosomes resolves discrepancy in time to common ancestor of males versus females. *Science* . doi:10.1126/science.1237619.

[96] Brotherton, P., Haak, W., Templeton, J., Brandt, G., Soubrier, J., Jane Adler, C., Richards, S. M., Sarkissian, C. D., Ganslmeier, R., Friederich, S., Dresely, V., van Oven, M., Kenyon, R., Van der Hoek, M. B., Korlach, J., Luong, K., Ho, S. Y. W., Quintana-Murci, L., Behar, D. M., Meller, H., Alt, K. W., Cooper, A., Adhikarla, S., Ganesh Prasad, A. K., Pitchappan, R., Varatharajan Santhakumari, A., Balanovska, E., Balanovsky, O., Bertranpetit, J., Comas, D., Martínez-Cruz, B., Melé, M., Clarke, A. C., Matisoo-Smith, E. A., Dulik, M. C., Gaieski, J. B., Owings, A. C., Schurr, T. G., Vilar, M. G., Hobbs, A., Soodyall, H., Javed, A., Parida, L., Platt, D. E., Royyuru, A. K., Jin, L., Li, S., Kaplan, M. E., Merchant, N. C., John Mitchell, R., Renfrew, C., Lacerda, D. R., Santos, F. R., Soria Hernanz, D. F., Spencer Wells, R., Swamikrishnan, P., Tyler-Smith, C., Paulo Vieira, P., Ziegle, J. S., and Consortium, T. G. (2013). Neolithic mitochondrial haplogroup H genomes and the genetic origins of Europeans. *Nature Communications* **4**(1), 1764. doi:org/10.1038/ncomms2656.

[97] Rieux, A., Eriksson, A., Li, M., Sobkowiak, B., Weinert, L. A., Warmuth, V., Ruiz-Linares, A., Manica, A., and Balloux, F. (2014). Improved Calibration of the Human Mitochondrial Clock Using Ancient Genomes. *Molecular Biology and Evolution* **31**(10), 2780–2792. doi:org/10.1093/molbev/msu222.

[98] Fu, Q., Li, H., Moorjani, P., Jay, F., Slepchenko, S. M., Bondarev, A. A., Johnson, P. L. F., Aximu-Petri, A., Prüfer, K., de Filippo, C., Meyer, M., Zwyns, N., Salazar-García, D. C., Kuzmin, Y. V., Keates, S. G., Kosintsev, P. A., Razhev, D. I., Richards, M. P., Peristov, N. V., Lachmann, M., Douka, K., Higham, T. F. G., Slatkin, M., Hublin, J.-J., Reich, D., Kelso, J., Viola, T. B., and Pääbo, S. (2014). Genome sequence of a 45,000-year-old modern human from western Siberia. *Nature* **514**(7523), 445–449. doi:org/10.1038/nature13810.

[99] Kivisild, T. (2015). Maternal ancestry and population history from whole mitochondrial genomes. *Investigative Genetics* **6**(1), 3. doi:org/10.1186/s13323-015-0022-2.

[100] Rambaut, A., Drummond, A. J., Xie, D., Baele, G., and Suchard, M. A. (2018). Posterior Summarization in Bayesian Phylogenetics Using Tracer 1.7. *Systematic Biology* **67**(5), 901–904. doi:10.1093/sysbio/syy032.

[101] Xu, S., Li, L., Luo, X., Chen, M., Tang, W., Zhan, L., Dai, Z., Lam, T. T., Guan, Y., and Yu, G. (2022). Ggtree: A serialized data object for visualization of a phylogenetic tree and annotation data. *iMeta* **1**(4), e56. doi:10.1002/imt2.56.

[102] Mitchell, P. and Lane, P. (2013). *The Oxford Handbook of African Archaeology.* Oxford University Press.

[103] Quintana-Murci, L., Harmant, C., Quach, H., Balanovsky, O., Zaporozhchenko, V., Bormans, C., van Helden, P. D., Hoal, E. G., and Behar, D. M. (2010). Strong

Maternal Khoisan Contribution to the South African Coloured Population: A Case of Gender-Biased Admixture. *American Journal of Human Genetics* **86**(4), 611–620. doi:10.1016/j.ajhg.2010.02.014.

[104] Boris Malyarchuk, Miroslava Derenko, M. P. and Vanecek, T. (2008). Mitochondrial Haplogroup U2d Phylogeny and Distribution. *Human Biology* **80**(5), 565–571. doi:10.3378/1534-6617-80.5.565.

[105] Barbieri, C., Vicente, M., Oliveira, S., Bostoen, K., Rocha, J., Stoneking, M., and Pakendorf, B. (2014). Migration and interaction in a contact zone: mtDNA variation among Bantu-speakers in Southern Africa. *PLoS ONE* **9**(6), 1–14. doi:10.1371/journal.pone.0099117.

[106] Harich, N., Costa, M. D., Fernandes, V., Kandil, M., Pereira, J. B., Silva, N. M., and Pereira, L. (2010). The trans-Saharan slave trade - clues from interpolation analyses and high-resolution characterization of mitochondrial DNA lineages. *BMC Evol Biol* **10**(1), 138. doi:10.1186/1471-2148-10-138.

[107] Cerezo, M., Achilli, A., Olivieri, A., Perego, U. A., Gómez-Carballa, A., Brisighelli, F., Lancioni, H., Woodward, S. R., López-Soto, M., Carracedo, Á., Capelli, C., Torroni, A., and Salas, A. (2012). Reconstructing ancient mitochondrial DNA links between Africa and Europe. *Genome Res* **22**(5), 821–826. doi:10.1101/gr.134452.111.

[108] Gomez, F., Hirbo, J., and Tishkoff, S. A. (2014). Genetic variation and adaptation in Africa: Implications for human evolution and disease. *Cold Spring Harb Perspect Biol* **6**(7), a008524. doi:10.1101/cshperspect.a008524.

[109] Fernandes, V., Triska, P., Pereira, J. B., Alshamali, F., Rito, T., Machado, A., Fajkošová, Z., Cavadas, B., Černý, V., Soares, P., Richards, M. B., and Pereira, L. (2015). Genetic Stratigraphy of Key Demographic Events in Arabia. *PLoS ONE* **10**(3), 1–27. doi:10.1371/journal.pone.0118625.

[110] Kivisild, T., Reidla, M., Metspalu, E., Rosa, A., Brehm, A., Pennarun, E., Parik, J., Geberhiwot, T., Usanga, E., and Villems, R. (2004). Ethiopian mitochondrial DNA heritage: tracking gene flow across and around the gate of tears. *Am J Hum Genet* **75**(5), 752–770. doi:10.1086/425161.

[111] Gandini, F., Achilli, A., Pala, M., Bodner, M., Brandini, S., Huber, G., Egyed, B., Ferretti, L., Gómez-Carballa, A., Salas, A., Scozzari, R., Cruciani, F., Coppa, A., Parson, W., Semino, O., Soares, P., Torroni, A., Richards, M. B., and Olivieri, A. (2016). Mapping human dispersals into the Horn of Africa from Arabian Ice Age refugia using mitogenomes. *Scientific Reports* **6**(1), 25472. doi:10.1038/srep25472.

[112] Kutanan, W., Kampuansai, J., Srikummool, M., Kangwanpong, D., Ghirotto, S., Brunelli, A., and Stoneking, M. (2017). Complete mitochondrial genomes of Thai and Lao populations indicate an ancient origin of Austroasiatic groups and demic diffusion in the spread of Tai-Kadai languages. *Hum Genet* **136**(1), 85–98. doi:10.1007/s00439-016-1742-y.

[113] Álvarez-Iglesias, V., Mosquera-Miguel, A., Cerezo, M., Quintáns, B., Zarrabeitia, M. T., Cuscó, I., Lareu, M. V., García, Ó., Pérez-Jurado, L., Carracedo, Á., and Salas, A. (2009). New Population and Phylogenetic Features of the Internal Variation within Mitochondrial DNA Macro-Haplogroup R0. *PLoS ONE* **4**(4), 1–9. doi:10.1371/journal.pone.0005112.

[114] Pala, M., Olivieri, A., Achilli, A., Accetturo, M., Metspalu, E., Reidla, M., Tamm, E., Karmin, M., Reisberg, T., Hooshiar Kashani, B., Perego, U. A., Carossa, V., Gandini, F., Pereira, J. B., Soares, P., Angerhofer, N., Rychkov, S., Al-Zahery, N., Carelli, V., Sanati, M. H., Houshmand, M., Hatina, J., Macaulay, V., Pereira, L., Woodward, S. R., Davies, W., Gamble, C., Baird, D., Semino, O., Villems, R., Torroni, A., and Richards, M. B. (2012). Mitochondrial DNA signals of late glacial recolonization of Europe from near eastern refugia. *Am J Hum Genet* **90**(5), 915–924. doi:10.1016/j.ajhg.2012.04.003.

[115] Costa, M. D., Pereira, J. B., Pala, M., Fernandes, V., Olivieri, A., Achilli, A., Perego, U. A., Rychkov, S., Naumova, O., Hatina, J., Woodward, S. R., Eng, K. K., Macaulay, V., Carr, M., Soares, P., Pereira, L., and Richards, M. B. (2013). A substantial prehistoric European ancestry amongst Ashkenazi maternal lineages. *Nat Commun* **4**, 2543. doi:10.1038/ncomms3543.

[116] González, A. M., García, O., Larruga, J., and Cabrera, V. M. (2006). The mitochondrial lineage U8a reveals a Paleolithic settlement in the Basque country. *BMC Genomics* **7**, 124. doi:10.1186/1471-2164-7-124.

[117] Ottoni, C., Primativo, G., Hooshiar Kashani, B., Achilli, A., Martínez-Labarga, C., Biondi, G., Torroni, A., and Rickards, O. (2010). Mitochondrial Haplogroup H1 in North Africa: An Early Holocene Arrival from Iberia. *PLoS ONE* **5**(10), 1–7. doi:10.1371/journal.pone.0013378.

[118] Secher, B., Fregel, R., Larruga, J., Cabrera, V. M., Endicott, P., Pestano, J., and González, A. M. (2014). The history of the North African mitochondrial DNA haplogroup U6 gene flow into the African, Eurasian and American continents. *BMC Evol Biol* **14**(1), 109. doi:10.1186/1471-2148-14-109.

[119] Hill, C., Soares, P., Mormina, M., Macaulay, V., Clarke, D., Blumbach, P. B., Vizuete-Forster, M., Forster, P., Bulbeck, D., Oppenheimer, S., and Richards, M. (2007). A mitochondrial stratigraphy for island southeast Asia. *Am J Hum Genet* **80**(1), 29–43. doi:10.1086/510412.

[120] Maji, S., Krithika, S., and Vasulu, T. S. (2009). Phylogeographic distribution of mitochondrial DNA macrohaplogroup M in India. *J Genet* **88**(1), 127–139. doi:10.1007/s12041-009-0020-3.

[121] Kumar, S., Ravuri, R. R., Koneru, P., Urade, B. P., Sarkar, B. N., Chandrasekar, A., and Rao, V. R. (2009). Reconstructing Indian-Australian phylogenetic link. *BMC Evol Biol* **9**, 173. doi:10.1186/1471-2148-9-173.

[122] Metspalu, M., Kivisild, T., Metspalu, E., Parik, J., Hudjashov, G., Kaldma, K., Serk, P., Karmin, M., Behar, D., Gilbert, M., Endicott, P., Mastana, S., Papiha,

S., Skorecki, K., Torroni, A., and Villems, R. (2004). Most of the extant mtDNA boundaries in South and Southwest Asia were likely shaped during the initial settlement of Eurasia by anatomically modern humans. *BMC Genetics* **5**, 26. doi:10.1186/1471-2156-5-26.
